# Ultrafast infrared observation of exciton equilibration from oriented single crystals of photosystem II

**DOI:** 10.1038/ncomms13977

**Published:** 2016-12-23

**Authors:** Marius Kaucikas, Karim Maghlaoui, Jim Barber, Thomas Renger, Jasper J. van Thor

**Affiliations:** 1 Imperial College London, South Kensington Campus, Sir Ernst Chain Building, London SW7 2AZ, UK; 2 Johannes Kepler University Linz, Institute of Theoretical Physics, Altenberger Str. 69, AT-4040 Linz, Austria

## Abstract

In oxygenic photosynthesis, two photosystems work in series. Each of them contains a reaction centre that is surrounded by light-harvesting antennae, which absorb the light and transfer the excitation energy to the reaction centre where electron transfer reactions are driven. Here we report a critical test for two contrasting models of light harvesting by photosystem II cores, known as the trap-limited and the transfer-to-the trap-limited model. Oriented single crystals of photosystem II core complexes of *Synechococcus elongatus* are excited by polarized visible light and the transient absorption is probed with polarized light in the infrared. The dichroic amplitudes resulting from photoselection are maintained on the 60 ps timescale that corresponds to the dominant energy transfer process providing compelling evidence for the transfer-to-the-trap limitation of the overall light-harvesting process. This finding has functional implications for the quenching of excited states allowing plants to survive under high light intensities.

## Abstract (web summary)

There are two common models of photosynthetic light harvesting by photosystem II cores. Here, the authors use structurally sensitive, time resolved infrared pleochroism of single crystals of photosystem II core complexes to probe the exciton equilibration that discriminates between the models.

Natural solar energy conversion by the oxygenic photosynthesis apparatus is very efficient to generate the initial charge separated state^1^,^2^. In oxygenic photosynthesis, the initial charge separation and electron transfer in photosystem II (PSII) results in water oxidation and oxygen evolution in the oxygen evolving complex, thus sustaining life on earth. Both energy transfer and charge separation in photosynthesis are very fast events that occur with very high quantum efficiency. The underlying electronic and structural dynamics occurring on ultrafast and longer timescales are also very robust to support efficient energy conversion under very different conditions of illumination. This is experimentally demonstrated in this contribution through comparison of the product amplitudes with directional photoselection of very different initial photoexcited populations.

The research of energy and charge dynamics in the PSII has been aided by the availability of high-resolution X-ray structures^3^,^4^,^5^,^6^,^7^. Recently, time-resolved X-ray crystallography has also probed structural dynamics following electron transfer, using the serial femtosecond crystallography method^8^. Exciton dynamics and charge transfer reactions, however, require spectroscopic probes, and there is a particular need for demonstrating structurally sensitive ultrafast spectroscopic measurements of these processes in natural energy conversion.

In the case of core complexes of PSII of *Synechococcus elongatus*, 74 chlorin pigments contribute to transient absorption signals during energy-transfer and charge-separation stages. This large number presents a problem of spectral congestion when analyzing time-resolved spectroscopic measurements of isotropic solutions of PSII. Typically, data processing uses target analysis and multicompartment analysis methods^9^,^10^,^11^. Such advanced kinetic modelling analyses the spectral dynamics and provides time domain information but lacks structural information^11^. Alternative nonlinear spectroscopic approaches retrieve coherence and frequency–frequency correlations for coupled pigments, from which structural pathways for energy transfer have been inferred, using structure-based theory^12^. Extension of time-domain dispersive measurements, carried out in this contribution in the mid-infrared spectral region, allows structural analysis from the macroscopic ordering.

According to the trap-limited model, exciton equilibration in the whole PSII core complex (PSIIcc) is fast and reversible electron transfer reactions determine the slow decay time of fluorescence. In contrast, in the transfer to the trap-limited model this decay time reflects slow excitation energy transfer between the core antennae and the reaction centre (RC), where the excitation energy is irreversibly trapped. Theoretical structure-based analysis of PSII ultrafast spectroscopy must use knowledge of the specific site energies of the individual pigments in addition to explicit theory that treats light-harvesting dynamics from first principles^13^,^14^. Based on such modelling, it has been suggested that the overall bottleneck of light-harvesting in PSIIccs is the transfer from the CP43 and CP47 subunits to the RC, where for open RCs the excitations are irreversibly trapped by primary electron transfer^14^. For closed RCs (having a reduced quinone *Q*_A_), the primary electron transfer reaction is slowed down such that about two-third of excitons escape the RC and are transferred to the low energy states of the antennae. This escape is promoted by the large entropy of excited antenna states, which is simply a result of the larger number of pigments in the CP43 and CP47 subunits as compared with the RC and the similar site energies of the pigments. Hence, a switch occurs from a light-harvesting mode at low light intensities to a photoprotective mode at high light intensities. In the latter case, excitons equilibrate between CP43 and CP47 via the RC on a 50 ps timescale, whereas for open RCs, CP43 and CP47 are disconnected, because excitons are trapped in the RC by ultrafast electron transfer^14^.

There are, however, alternative interpretations of kinetic data, suggesting that, instead of the transfer to the trap, the charge transfer is overall rate limiting in PSII^15^,^16^. The latter model is known in the literature as excited-state radical pair equilibrium (ERPE) model. In this model, the dominating ∼40–60 ps time constant for the decay of excited states in PSIIccs with open RCs was assigned to reversible electron transfer, assuming that exciton equilibration in the whole core complex occurs on a 1.5 ps timescale^14^,^16^. Hence, there is more than an order of magnitude difference in excitation energy transfer times between the two models of light-harvesting in PSIIccs. The present technique is ideally suited to solve this problem, as ultrafast exciton equilibration should erase any orientational information that is prepared by the polarization of the pump pulse in the oriented complexes in the crystal.

## Results

### Laboratory orientation of orthorhombic PSII core crystals

The index of oriented single PSII crystals was determined using synchrotron X-ray diffraction measurements. All of the tested crystals had cuboid morphology with one dimension being significantly smaller than the other two. Indexing of 17 crystals determined that for all but one the shortest dimension could unequivocally be assigned to the *a* axis direction and the spectroscopically accessible face corresponded to the {1 0 0} plane. Moreover, the crystals with rectangular plate morphology had their longest axis coinciding with either *b* or *c* axis. This type of morphology was chosen for polarized infrared (IR) measurements of the oriented crystals^17^, and the consequent spectroscopic results confirmed the conclusion of X-ray indexing. Analysis of the data from 11 rectangular plate morphology crystals identified spectral features that divide the crystals in two distinct classes. These features were used to determine the direction of *c* and *b* axes (See Supplementary Methods for details.)

### Polarized fs IR measurements on oriented single PSII crystals

The femtosecond transient infrared pleochroism measurements of PSII crystals^18^ were performed with visible excitation (680 nm, 100 fs, 1 W cm^−2^, 1 kHz, spot diameter 200 μm, approximately 2.5 photons per core complex) polarized along either of the two longest crystal edges, with PSII in the closed state. As discussed above, this corresponded to propagation along the crystallographic *a* axis and polarization coinciding with *b* or *c* axis. The probing light (6 μm, 100 fs, 180 cm^−1^ bandwidth) was much weaker and was polarized either parallel or orthogonal relative to the excitation. Thus, for each crystal, four time-resolved difference spectra were acquired at every delay point. The delay between pump and probe pulses was varied from 0.1 to 1500, ps in randomized manner, and the data were checked after each scan for the signs of sample degradation. The acquired data were processed using Singular Value Decomposition and Global Analysis methods (see Supplementary Methods for details, Supplementary Fig. 14 for Global Analysis results). For all polarization combinations, the analysis consistently indicated the presence of three time constants, with representative values of 3, 61.5 and 832 ps. Careful analysis could not resolve subpicosecond processes, in part due to the available instrument response and time zero coherent artefacts. The multiexponential kinetics results from a mixture of contributing processes. Because also each kinetic phase represents a mixture of contributing signals, target analysis has previously been applied to model time-resolved measurements of solution samples of PSII cores, which analyses contributions from the CP43, CP47 and RC ‘compartments' as linear combinations^19^. Here we restrict our analysis to describing the dominant physical processes of the smallest number of time constants that can globally fit the data. The 3 ps time constant describes the decay of the initial photoexcited population and represents exciton equilibration among the pigments within the CP43, CP47 and RC subunits^14^. Because of the high light intensities applied with an estimated 2.5 absorbed photons per core, we may also expect exciton-annihilation effects^20^. The 61.5 ps time constant corresponds primarily to energy-transfer processes between the subunits^11^,^14^ and the P680^+^/Pheo^−^ charge-separated state develops during the 832 ps phase corresponding to the average time constant of 360 ps and 12 ns reported in ref. 19. As the P680^+^/Pheo^−^ difference spectrum only has contributions from two copies in the asymmetric unit, a specific structural measurement was possible on the basis of band-fitting, which was seen to fit the X-ray coordinates well using published vibrational mode assignments for the P680^+^/P680 and Pheo^−^/Pheo contributions (Supplementary Methods, Supplementary Figs 4, 13 and 16–20, Supplementary Tables 3 and 4).

**Figure 1:**
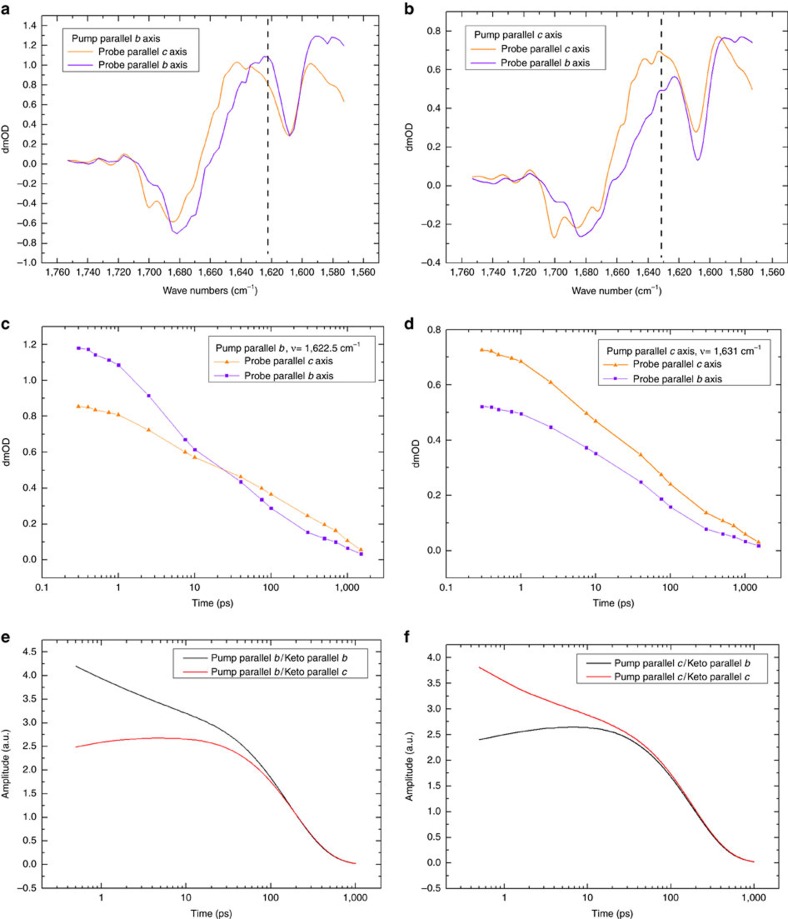
Directional photoselection and IR dichroic amplitude differences demonstrate macroscopic local mode character of excited-state keto modes. (**a**) Polarized 3 ps decay spectra for the *b* and *c* directions with optical excitation in the *b* direction. The dashed line indicates the 1622.5 cm^−1^ wavenumber shown in panel (**c**). (**b**) Polarized 3 ps decay spectra for the *b* and *c* directions with optical excitation in the *c* direction. The dashed line indicates the 1631, cm^−1^ wavenumber shown in panel (**d**). (**c**) Time traces for polarized signals at 1622.5 cm^−1^ in the *b* and *c* directions with optical excitation in the *c* direction. (**d**) Time traces for polarized signals at 1631, cm^−1^ in the *b* and *c* directions with optical excitation in the *b* direction. (**e**) Full simulation of infrared dichroic signals in the *b* and *c* directions with optical excitation in the *b* direction, summing all keto modes and assuming local mode vibrational character and applying simulated populations from theory. (**f**) Full simulation of infrared dichroic signals in the *b* and *c* directions with optical excitation in the *c* direction, summing all keto modes and assuming local mode vibrational character and applying simulated populations from theory.

Figure 1a,b shows the difference spectra of the 3 ps decay phase for two orthogonal pump polarizations coinciding with crystallographic *b* and *c* axes. It can be observed that there is a clear spectral difference between the photoselected initial populations, especially in the 1620–1700, cm^−1^ region. This region is dominated by chlorophyll *a* 13-1 keto C=O stretching modes with ground- and excited-state absorptions located in the 1670–1700 and 1620–1670, cm^−1^ regions correspondingly^19^.

The early time infrared dichroic amplitudes can provide evidence that the Chl *a* 13-1 keto C=O modes have a dominating local mode character, allowing subsequent analysis of infrared dichroism in the local basis from X-ray coordinate analysis. Both the visible *Q*_Y_ transition dipole moment (TDM) and the infrared active 13-1 keto C=O stretching TDM are in-plane modes. Their directions can be determined by time-dependent density functional theory (TD-DFT) and DFT harmonic frequency calculations, correspondingly. For isolated Chl *a*, the angle between these two TDM was calculated to be 14.7° (Supplementary Table 1). Thus the Chl *a* 13-1 keto C=O mode may act as a ‘projector' of the visible TDM and its dichroic ratio *R* will be strongly determined by the photoselection by polarized excitation. However, this can only be true if the Chl *a* 13-1 keto C=O mode can be considered as local mode, with multiple Chl *a* contributing to the sub-ps dichroic amplitudes. Full simulations of the optical crystallographic photoselection of initial populations and exciton transfer demonstrate this for the PSII dimer (Fig. 1e,f). Assuming a local mode character, the sum of all the isolated 13-1 keto C=O TDM of excited pigment populations under our experimental conditions (680 nm, 100 fs, *E*-field parallel to *b*; *E*-field parallel to *c*) are indeed predicted to have significant dependence on the direction of optical pumping and maximize in the direction of the visible pump polarization (Fig. 1e,f). Experimentally, it was found that the dominant excited-state transient absorption at 1622, cm^−1^ (pumping parallel to *b*; Fig. 1c) and 1631, cm^−1^ (pumping parallel to *c*; Fig. 1d) has a polarization dependence that is qualitatively comparable to the theoretical calculations (Fig. 1e,f). The correspondence between the experimental (Fig. 1c,d) and calculated (Fig. 1e,f) traces is further evaluated on the basis of details taken for site energies (Supplementary Fig. 12), limited contribution from exciton to exciton annihilation (Supplementary Figs 9–12, below) and small levels of vibrational mode mixing of the 13-1 keto C=O mode. The primary considerations are that photoselection amplitudes directly after excitation are directly comparable between experiment (Fig. 1c,d) and simulation (Fig. 1e,f) and that experimental infrared dichroic differences are maintained on the ∼10–60 ps timescale that is characteristic for the major energy-transfer processes, in agreement with theory (Fig. 1e,f, Supplementary Figs 9, 10 and 12). Inclusion of exciton–exciton annihilation processes in the calculation of the spectra are found to modify the shape of the decay curve but, to a large extent, maintain the dichroic ratio (the difference of the signal for the two probe polarizations) of the decay (Supplementary Figs 9, 10 and 12). Variations in site energies of the pigments^21^ can give rise to a crossing between the pump and probe curves as seen in the experiment for pump polarization parallel to the crystal *b* axis at a delay time of about 11 ps (Fig. 1c, Supplementary Fig. 12), but for longer delay times the dichroic ratio is not unity, as one would expect for very fast exciton transfer between antenna subunits owing to the complete randomization of the polarization (Supplementary Fig. 8).

It should be noted that the 1622 and 1631, cm^−1^ bands are dominant and should have contribution from many pigments, such that its polarization dependence may be compared qualitatively with the simulated sum of all keto modes. This qualitative correspondence is taken as very clear evidence that the keto C=O stretching modes can indeed be analysed as local modes, validating the analysis of infrared pleochroic measurements of exciton dynamics and charge separation in the local pigment basis.

For quantitative analysis of dichroic amplitudes, it was possible to model the 3 ps spectra assuming a minimum of three spectrally distinct populations of Chl *a*. It was further assumed that the direction of the Chl *a* 13-1 keto C=O mode TDM is the same for ground and excited states, thus fixing the dichroism for the bleach and transient bands. This model provided a good fit of the experimental spectra with the minimum number of parameters (see Supplementary Methods) The resulting fit of the experimental spectrum is also shown in Fig. 1a,b and the resulting bands are presented in Supplementary Fig. 15 and Supplementary Table 2.

The band-fitting analysis then allows the correspondence between experimental and theoretical TDM directions from their projections on the {1 0 0} plane. As described in the Methods section, the contribution *p*_*i*_ of each pigment to the experimental dichroic ratio is obtained from the dipole length-weighted inner products of the squared components in the *b* and *c* direction with the dichroic absorbances that are experimentally determined (equation 2). Therefore, for each of the three centre frequencies of the contributions *p*_*i*_ to the 3 ps spectrum, different values for *p*_*i*_ are found for each pigment *i*=1..74, which represent their contributions based on direction only. Similarly, with pumping in the *c* direction a new set of values is found, for each frequency.

**Figure 2:**
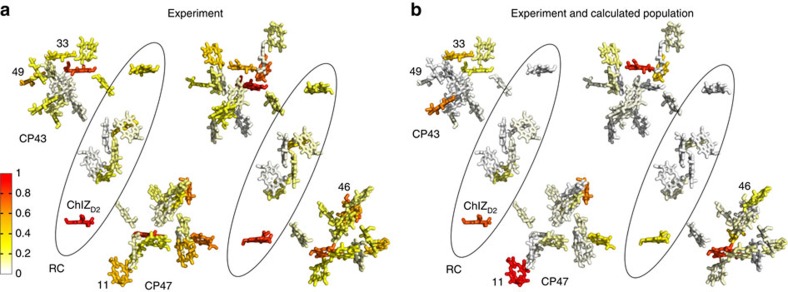
Frequency-selected fs IR pleochroism analysis and theory of excited-state populations in PSII cores in the single pigment basis from crystallographic coordinates. (**a**) Squared dipole projection *p*_*i*_ for each pigment contributing to the 1622(+)/1676(−) cm^−1^ bands in the 3 ps decay phase with optical excitation in the *b* direction (Methods section, equation 2). (**b**) Theoretical calculation using non-Markovian density matrix theory of initial photolysed population with excitation along the *b* axis multiplied with the squared dipole projections seen in panel (**a**). Selected chlorophylls are labeled with numbering as discussed in the text.

Structure-based exciton calculations were performed that used non-Markovian density matrix theory for the optical excitation^22^, Redfield theory for exciton relaxation within domains of strongly coupled pigments and generalized Förster theory for excitation energy transfer between these domains^14^,^23^,^24^. The site energies and excitonic couplings obtained in earlier work^25^ were used to parameterize the exciton Hamiltonian. Furthermore, the modelling was adapted to the crystallographic case by taking into account the photoselection by pump light polarized along one of the crystallographic axes. This was implemented using optical transition dipole vectors obtained using TD-DFT calculations and weighing each single exciton state by projection of its TDM on the corresponding polarization directions of the optical pump field that are parallel to the selected crystal axes. The calculations were performed for the full dimer present in the asymmetric unit and included the laser pulse central wavelength, bandwidth and polarization (Fig. 2a, Supplementary Methods).

The resulting excited-state populations were further weighed by the factor *p*_*i*_ (equation 2) that characterizes the single pigment contribution to the infrared absorbance of light polarized in the (1,0,0) plane, thus incorporating the information obtained from polarization-resolved infrared measurements. Figure 2b shows the predicted contribution of each Chl *a* molecule to the initial excited state based on calculations and 1622(+)/1676(−) cm^−1^ band dichroism for the case that the optical field is in the crystallographic *b* direction.

The differences between the quantities shown in Fig. 2a,b predict, for example, that in the 3 ps decay process Chl 46 (numbering according to Loll *et al*.^3^ and Shibata *et al*.^25^) of CP43 is likely to contribute to the 1622(+)/1676(−) cm^−1^ band (*p*_*i*_=0.46) based on direction alone (Fig. 1a) but is predicted to be only weakly populated (population N_46_(*t*=0)=0.028) directly after excitation according to theory (Fig. 1b). Note that the equivalent Chl46 in the second monomer (Chl 5500 in numbering according to Loll *et al*.^3^) has only low probability to contribute. Other pigments such as Chl 33 (Chl 5491 according to Loll *et al*.^3^) of CP43 do have a correspondence between the dichroic contribution (*p*_*i*_=0.66) and calculated population (*N*_5491_(*t*=0)=0.55), Interestingly, the corresponding pigment Chl 33 in the other dimer (Chl 491) also has a favourable keto TDM direction (*p*_*i*_=0.83) but a relatively reduced predicted population (*N*_491_(*t*=0)=0.16). Nevertheless, as an example it is shown that Chl 33 is likely to contribute to measurements at 1622(+)/1676(−) cm^−1^ at early delays with *b*-polarized pumping but much less so for *c*-polarized pumping. Similarly, the dichroic contributions *p*_*i*_ for each pigment may be calculated using equation 2 presented in the Method section and Supplementary Table 1 for each frequency for the 3 and 61.5 ps decay spectra with *b*- or *c*-excitation direction.

**Figure 3:**
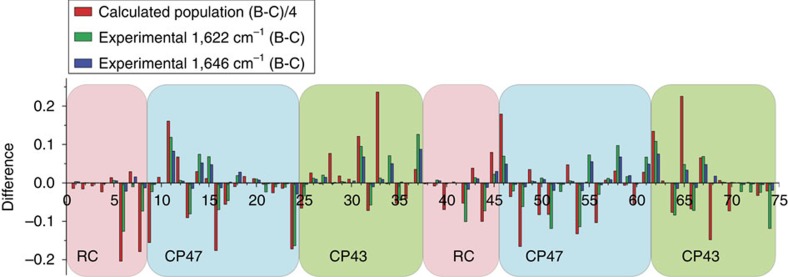
Selectivity analysis for photoselection differences of theoretical populations and experimental dichroic contributions. The red bars represent the differences in the calculated initial populations with optical excitation in the *b* minus the *c* directions, using non-Markovian density matrix theory only. The green bars are the differences in squared dipole projections *p*_*i*_ of the 1622(+)/1676(−) cm^−1^ dichroic contributions with excitation along *b* minus that of the *c* direction. The blue bars represent the differences in the squared dipole projections *p*_*i*_ for the 1646(+)/1687(−) cm^−1^ bands. Pigments are numbered as in Loll *et al*.^3^ and Shibata *et al*.^25^

The analysis in Fig. 2b considers the correspondence between vibrational TDM directions and calculated population as a first approach. An independent comparison between the predicted initial population from non-Markovian density matrix theory and the experimental dichroic ratio assess the differences with optical excitation in the crystallographic *b* and *c* directions. The directional selectivity of the dichroic contributions *p*_*i*_ were evaluated for the two strongest bands in the 13-1 keto C=O region of the 3 ps spectra at 1622(+)/1676(−) and 1646(+)/1687(−) cm^−1^. The differences for the dichroic amplitudes *p*_*i*_ may then be compared with the differences for the simulated initial populations taken from theory with pumping in either the *b* or *c* direction. Figure 3 shows the resulting polarization selectivity measures for all pigments in the PSIIcc. For example, in the case of the *c*-polarized pump, chlorophyll 15 of CP47 (nr. 13; Fig. 3) is more probable to contribute to the 1622(+)/1676(−) cm^−1^ band rather than the 1646(+)/1687(−) cm^−1^ band from the comparison with preferential population. On the other hand, ChlZ_D2_ (nr. 45; Fig. 3) is more likely to contribute to the 1646(+)/1687(−) cm^−1^ band.

The contribution of exciton annihilation resulting from the estimated 2.5 absorbed photons per core complex is evaluated on the basis of calculations that include up to 2 excitations of the core complex (Supplementary Figs 9–12) in addition to power density-dependence measurements. From these calculations, it is seen that exciton annihilation leads to somewhat faster decay of the pump–probe signal in the 2–20 ps time range but does not change the dichroic ratio appreciably. This timescale of the annihilation processes suggests that they predominantly occur between exciton domains located in the same antenna subunit, whereas the intersubunit transfer that dominates the decay of the dichroic ratio is not affected. We conclude that annihilation does not change the main conclusions based on evaluation of the singly excited-state dynamics (Supplementary Figs 9, 10 and 12). A more detailed discussion of the influence of exciton–exciton annihilation, including an estimate for the decrease in signal amplitude at large delay times is given in Supplementary Methods.

After initial excitation, equilibration and annihilation, the energy is equilibrating in the core complex on a 60 ps timescale. Owing to transfer to red pigments, non-crystallographic symmetry is gradually developed, as seen experimentally (Fig. 1c,d) and by simulation (Figs 1e,f and 2b, Supplementary Figs 3 and 5–10). This transfer dynamics was simulated using excitation parameters from the experiment (100 fs, 680 nm) for the *b*- and *c*-polarized pump radiation. The exciton simulation predicts that, in the case of *b*-polarized excitation, high initial populations for Chl37 of CP43 (nr. 65), Chl45 of CP43 (nr. 33) and Chl 11 of CP47 (nr. 46) are expected (Supplementary Fig. 3). These populations decay on the picosecond timescale. The simulations result in these red pigments (having site energies corresponding to wavelengths as long as 689.5 nm^25^) become populated on a comparable timescale. Namely, Chl 29 of CP47 in both copies (nrs. 24 and 61) are predicted to become transiently populated. The relative contribution of these pigments maximizes at ∼50–100 ps, which corresponds to the equilibration time of excitons between CP43 and CP47 through the closed RC^14^.

The time evolution of dichroic differences may be transformed to visualize the probability for energy transfer at later delays. Adjusting for the population decay, the relative population may be visualized by comparison of the distribution seen in Supplementary Fig. 5a at 0 fs to that at 60 ps (Supplementary Fig. 5b). To construct the distributions at 60 ps, the dichroic contributions *p*_*i*_ taken from the 61.5 ps decay spectra for pumping in the *b* direction were multiplied with the calculated populations at 60 ps and subsequently coloured by adjusting the scale to the largest values (which would be much weaker at the same scale used in Fig. 2b). This distribution shows the highest values for both copies of the red pigments Chl 29 of CP47 (nrs. 24 and 61), indicating that the dichroic contributions at 61.5 ps and 1619.5 cm^−1^ are in agreement with the expected populations and low site energies, as an example (Supplementary Fig. 5). In order to represent all excited-state measurements, the *p*_*i*_ values for all contributions are summed and graphically presented to highlight the re-distributions after 60 ps (Fig. 1e,f, Supplementary Figs 5, 6 and 7). By summation of domains Domain 1 (CP47-Chlz_D2_), Domain 2 (CP43-Chlz_D1_) Domain 3 (D1D2 minus Chlz), it is seen that equilibration involves re-distribution from CP43 to CP47 on this timescale (Supplementary Fig. 7).

The fact that the system loses memory of the initially prepared excited state on a 100 ps timescale provides compelling evidence for slow energy transfer between the different subunits, as proposed by the transfer-to-the-trap limited model of light harvesting^14^. There is no doubt that such slow decay of the polarization-dependent signature excludes the trap-limited model of light harvesting in PSIIccs^15^,^16^ that assumes exciton equilibration on a one to two orders of magnitude faster timescale. Supplementary Fig. 8 illustrates this by showing that, for a calculation that simulates the ERPE situation, the dichroic differences collapse within a picosecond of optical excitation.

## Discussion

Ultrafast polarized infrared measurements of oriented PSII crystals is a special case in which non-crystallographic symmetry of pleochroism is time dependent. Through photoselection, the initial populations of both copies differ at early delays while non-crystallographic symmetry is gradually developed after about 100 ps owing to the tendency to transfer energy to red pigments on these timescales. The infrared measurements of the exciton-annihilation and energy-transfer stages of photosynthesis show that *b*- and *c*-polarized excitation results in very different excited-state populations and the time-dependent analysis of the dichroic amplitudes are possible from the discovery of the local mode character of the vibrational TDMs of the keto C=O stretching mode of Chl *a*. The measurements also demonstrate the flexibility of the light-harvesting and charge-separation processes that enable electron transfer with very high quantum efficiency in both cases. Without structural information, it would not be possible to distinguish between the two light-harvesting/charge-transfer models solely from the fluorescence decay. In contrast, the calculation of visible pump–infrared probe spectra in the middle and bottom part of Supplementary Fig. 8 reveals significant differences of the two models. Owing to the faster exciton transfer in the ERPE model, the system ‘forgets' the polarization of the initial excitation much faster than in the structure-based transfer-to-the-trap-limited model. Consequently, the anisotropy of the pump–probe signal is practically absent in the ERPE model but clearly visible in the structure-based model. By comparing the calculated with the measured pump–probe signals (Fig. 1c,d), it becomes clear that only the structure-based transfer to the trap-limited model can at least qualitatively explain the experimental data. The present experiments provide the closest approach to visualize exciton relaxation in time and space so far. Convincing evidence is obtained for 50–100 ps exciton equilibration between CP43 and CP47 subunits across the intervening RC in PSIIccs for closed RCs. In the latter case, excitation energy is proposed to accumulate at Chl 29 in CP47 (refs 14, 25) or at Chl 11 (ref. 21), which are situated at the periphery of the complex and might represent photoprotective sites, where the excitation energy is quenched. In the case of open RCs, excitons do not equilibrate between CP43 and CP47 but are trapped by electron transfer in the RC^14^. The bottleneck, however, remains the same, namely, transfer between the core antennae and the RC. As noted before, this slow transfer allows the RC to trap every exciton arriving in the RC by ultrafast electron transfer. The switch of the core complex into the photoprotective mode for closed RCs is triggered by the slowing down of primary charge transfer and promoted by the large entropy of excited antenna states that gives rise to a factor three larger rate constant of energy transfer from the RC to the antenna subunits than vice versa^14^.

We note that the present results are also in agreement with a recent structure-based theoretical study^26^ on light harvesting in PSII supercomplexes. In this study, an effective time constant of 100 ps has been inferred for transfer between the core antennae and the RC and an effective time constant of 110 ps for exciton diffusion in the whole super complex, containing peripheral light-harvesting complexes besides the core antennae^26^. Also, in the latter study, it was emphasized that a fit of time-resolved fluorescence data by kinetic models, without taking into account structural details, can lead to ambiguous conclusions about mechanistic details of the light-harvesting reaction. The present experiment removes this ambiguity for PSIIccs.

## Methods

### PSII Crystals

20 l cultures of wild-type *Thermosynechococcus elongatus* were grown in DTN-medium at 57 °C while bubbling continuously with 5% CO_2_-enriched air and illuminating with white light (Growlux, Sylvania) of increasing intensity (50–250 microeinsteins) according to cell density. Cells were harvested in the log phase after 8 days at a final OD750 of about 1.0. Thylakoid membranes were prepared as described by ref. 27 and PSIIccs isolated as previously reported^28^ using two successive anion-exchange chromatography steps^29^. Dimeric PSIIcc were concentrated in Vivaspin 20 centrifugal concentrators (Sartorius, UK) at 4 °C and 3,000*g*, (GH-3.8 rotor, Beckman Coulter Ltd., UK). Freshly isolated dimeric PSIIcc aliquots were either used for crystallization or frozen away after addition of glycerol to a concentration of 25%.

The vapour diffusion hanging drop method was used with presiliconized cover slides (Hampton Research, USA) and 24-well pregreased VDX plates (Hampton Research, USA). Crystallization conditions were based on previously reported results^4^ except for the following modifications. All buffers from now on were prepared with deuterium oxide instead of water and PSIIcc aliquots were washed as follows. Typically 50 μl at 4 mg ml^−1^ were washed with five cycles of fivefold dilution and concentration with deuterium oxide buffer (40 mM MES-NaOH pH 6.0, 10 mM CaCl_2_, 10 mM MgCl_2_, 5 % (w/w) glycerol, 0.02 % (w/v) β-DDM) using Vivaspin 500 centrifugal concentrators (100,000 MWCO, Sartorius Stedim UK Ltd) to remove traces of water. The Chl *a* concentration was adjusted to 2.0 mg ml^−1^ with the same buffer and the sample was centrifuged for 30 min at 4 °C and 22,000*g* (22R microfuge, Beckman Coulter Ltd., UK) in a 0.5 ml Eppendorf tube to remove aggregates. In all, 1–3 μl of mother liquor (100 mM Hepes-NaOH pH 7.5, 100 mM (NH_4_)2SO_4_, 8–10% PEG 4000, 20 % (w/w) glycerol, 1 mM heavy atom derivative, 0.11 mM polyoxyethylene lauryl ether (C12E8) twice as concentrated as the reservoir solution) was added in a 1:1 ratio with a solution of dimeric PSIIcc in buffer (20 mM MES-NaOH pH 6.5, 10 mM MgCl_2_, 10 mM CaCl_2_, 5 % (w/w) glycerol). The heavy atom derivatives used was Methyl Mercurial Chloride (Hampton Research, USA). The presiliconized cover slides (Hampton Research, USA) were then placed and sealed on top of wells containing 500 μl of 4–5 % PEG 4000 before placing the tray in a vibration-free incubator at 17 °C. Small square-plate crystals appeared within 24 h and were used in the experiment. All measurements were performed at room temperature.

### Micro-spectrometer

The experimental setup was as described before^18^. Briefly, the output of Ti:Sapphire regenerative amplifier (Spitfire PRO, Spectra Physics, 4 W, 70 fs) was divided between two optical parametric amplifiers (Topas-C, Light Conversion). One of the parametric amplifiers was equipped with noncolinear difference frequency generation module that produced mid-infrared probe pulses. The other used additional frequency mixing stages to generate visible pump pulses. The mid-infrared probe beam was directed to the polarization modulator that produced a sequence of orthogonally polarized pulses. This beam was then split into two to obtain signal and reference beams. The reference beam did not pass the sample but was directed to the reference spectrometer. The signal beam was directed into a infrared microscope (HYPERION 1000, Bruker) equipped with reflective objectives (36 × , NA=0.5). As it was reported previously^18^, the beam spot at the focus of the microscope focus was around 10 μm depending slightly on the alignment. The polarization of the probe beam at the focus was confirmed using wire grid polarizer. At the output of the microscope, the beam was collimated and directed to the signal spectrometer. The pump beam was directed (1 W cm^−2^) to the sample using a right angle prism located in the blind spot of the reflective objective. The beam was focussed on the sample to approximately 200 μm spot. Its intensity was adjusted using reflective neutral density filters to a level that allowed repetitive measurements on the same sample without significant degradation (see Supplementary Methods and Supplementary Figs 21 and 22). The pump beam wavelength was 680 nm in all measurements reported in this paper. The polarization of the pump beam was controlled by zero-order half-wave plate and coincided with either of the probe beam polarizations. Detection system consisted of two spectrometers (Triax190, Horiba) with mercury cadmium telluride array detectors (128 pixel arrays, Infrared Systems Development Corp.) attached to them. The measurements were performed at 6.1 μm centre probe wavelength and the spectrometer resolution of 3.3 cm^−1^. Control of the experiment, data collection and preprocessing was performed with the custom software written in LabVIEW.

### Data Processing

The polarization-resolved spectra and their fitting results carry structural information (Sage *et al*.^17^). For orthorhombic crystals of the PSII cores, the experimental measurements on the {1 0 0} face provide the absorbances *A*_*b*_ and *A*_*c*_ with polarizations along crystallographic *b* and *c* axes of the oriented single crystals. Following the notation of Sage *et al*.^17^, we define a unit vector parallel to TDM as *μ*_*i*_ (where *i*=1…*N*, *N*—the number of molecules) and its projections on *b* and *c* axes correspondingly as *μ*_*ib*_ and *μ*_ic_. To evaluate the relative contribution of each molecule to the absorption, we introduce an effective TDM *μ*_E_ that represents superposition of all individual absorption contributions. Then the experimentally measured dichroic ratio *R* between *c*-polarized probe beam absorption (*A*_c_) and *b*-polarized probe beam absorption (*A*_*b*_) is equal to (see also Supplementary Figs 1 and 2):


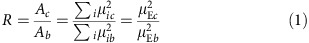


Here *μ*_E*b*_ and *μ*_E*c*_ are the projections of **μ**_E_ on *b* and *c* axes correspondingly. The squared magnitude of the effective vector **μ**_E_ is 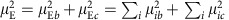. Then, for measurements on the {1 0 0} plane, the relative contribution of each individual molecule is proportional to the scalar product of the squared dipole projection in the *b*–*c* plane, *μ*_*ibc*_, with the absorbance vector **A**_*bc*_=(*A*_*b*_, *A*_*c*_)


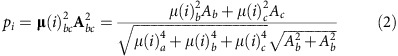


The relative contributions of each pigment *C*_*i*_ equals the ratio of *p*_*i*_ and the sum of all *p*_*i*_,


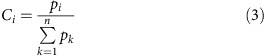


but the equation above are normalized to the maximal possible vector projection for convenience. This projection furthermore incorporates the ambiguity for the two possible dipole directions (2π−*ϕ* and *ϕ*) for the azimuthal angle *ϕ* that results in the same values for *R* and *p*_*i*_ owing to taking the square of the dipole component (Sage *et al*.^17^).

Harmonic frequency calculation using DFT was used to calculate the TDM direction of the Chl *a* 13-1 keto C=O stretching modes relative to the crystallographic axes. The TDM vectors were subsequently rotated to all 74 pigments in the X-ray crystal structure. The resulting of *μ*_*ib*_ and *μ*_*ic*_ values (see Supplementary Table 2) were used to obtain contribution of each Chl *a* to the 3 ps absorption bands. For example, in the case of 1622(+)/1676(−) cm^−1^ band, which represents 33% of the 3 ps decay amplitude for pumping in the *b* direction, the relative contribution of each Chl *a* molecule is presented in Fig. 1a.

### Data availability

The data that support the findings of this study are available from the corresponding author upon reasonable request.

## Additional information

**How to cite this article:** Kaucikas, M. *et al*. Ultrafast infrared observation of exciton equilibration from oriented single crystals of photosystem II. *Nat. Commun.* **7,** 13977 doi: 10.1038/ncomms13977 (2016).

**Publisher's note:** Springer Nature remains neutral with regard to jurisdictional claims in published maps and institutional affiliations.

## Supplementary Material

Supplementary Information Supplementary Figures, Supplementary Tables, Supplementary Methods and Supplementary References.
