# Supplementary material for: Ultrafast infrared observation of exciton equilibration from oriented single crystals of photosystem II
Source: Nat Commun. 2016 Dec 23;7:13977. doi: 10.1038/ncomms13977 (PMC5196431; doi:10.1038/ncomms13977)
Supplement: Supplementary Information — Supplementary Figures, Supplementary Tables, Supplementary Methods and Supplementary References. [file ncomms13977-s1.pdf]

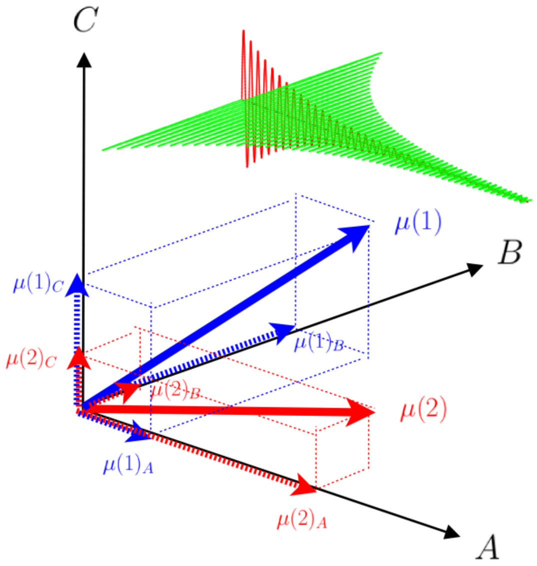

Supplementary Figure 1. Vector presentation example for *two* dipoles μ(1) and μ(2) placed in the asymmetric unit of a crystal with orthorhombic symmetry and their projections on the A, B and C axes. For a ray with a k vector parallel to the A axis, the allowed orthogonal polarisation directions E1 and E2 are wavelength independent, are associated with refractive indices n1 and n2 and experience attenuation coefficients proportional to $(\mu(1)_B)^2+(\mu(2)_B)^2$ and $(\mu(1)_C)^2+(\mu(2)_C)^2$.

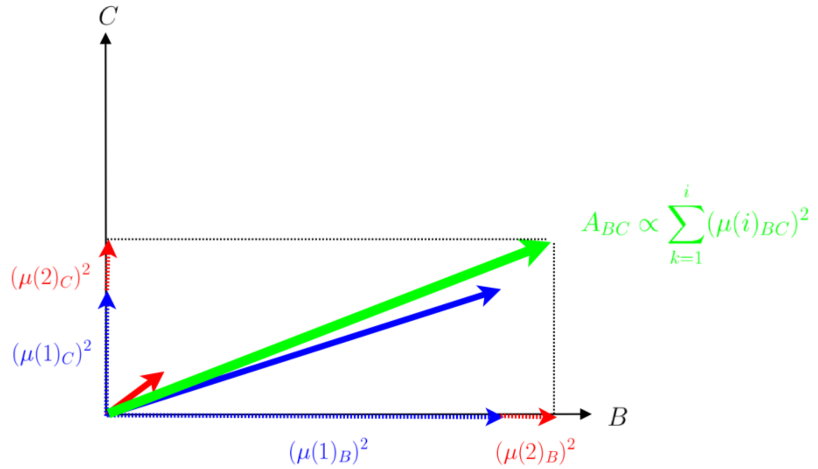

Supplementary Figure 2. Vector representation example demonstrating the resulting dichroic absorbances for *two* dipoles μ(1) and μ(2) placed in the geometry shown in Supplementary Figure 1. The contributions to the measured linear response are proportional to the vector projections $(\mu(i)_{BC})^2$ of the contributing dipoles onto the resulting dichroic absorbance vector A_{BC} (equation in Methods section of the main text).

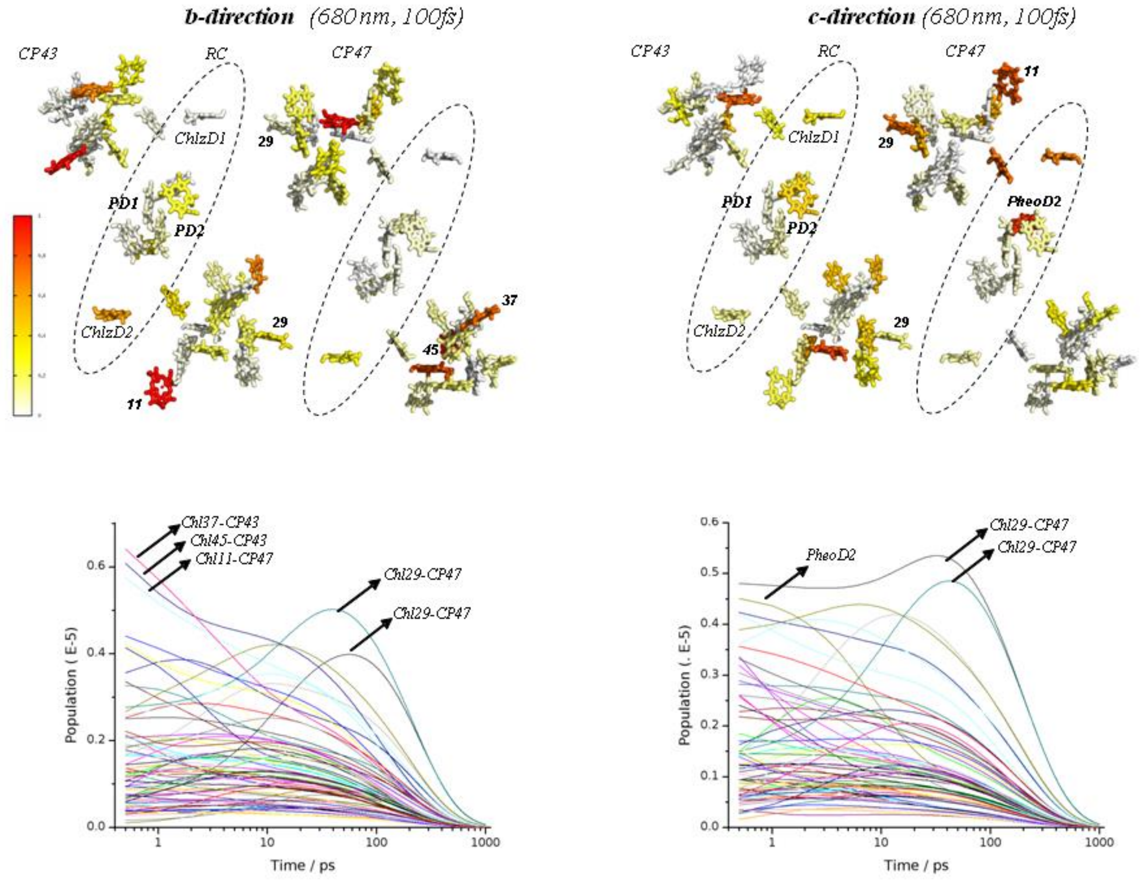

Supplementary Figure 3. Calculated populations $N_i(t)$ at time zero using non-Markovian density matrix theory with 680nm, 100fs pulses and polarisation in either the b- (left) or c- direction) are shown using white-yellow-orange-red colour scheme (top). The corresponding time traces for b- and c- excitation are shown in the traces below.

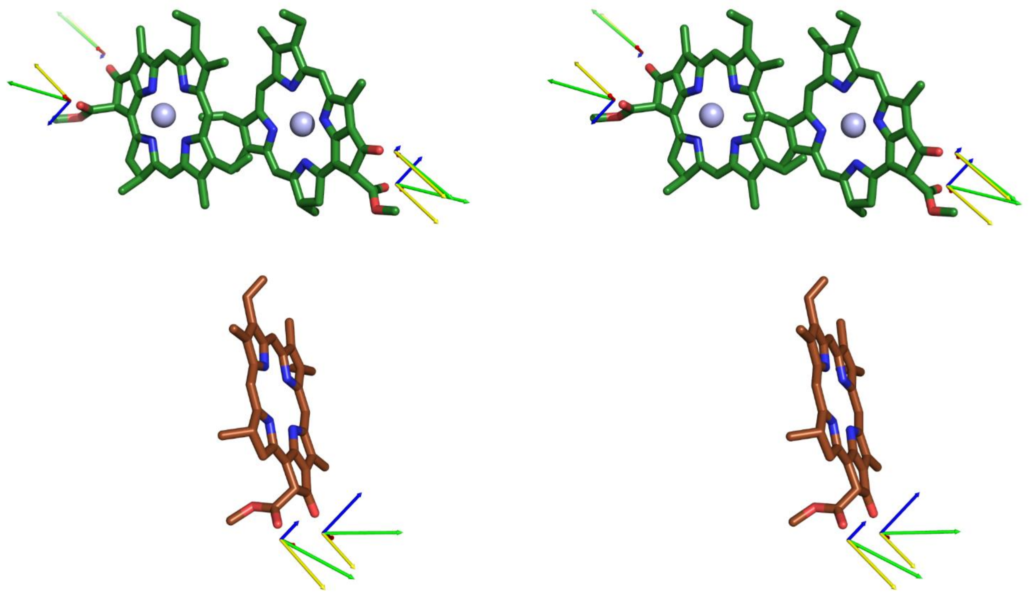

Supplementary Figure 4**.** A.Stereo figure of the 13-1 keto and ester TDM of the P680+/Pheo- C=O stretching modes. Shown are the non-crystallographic sums of squared TDM projections $\Sigma(\mu.a)^2$ (yellow) , $\Sigma\ (\mu.a)^2$ (red) and $\Sigma\ (\mu.c)^2$ (blue) and the $\Sigma\mu$ (green), see Supplementary Table 1

A: Time zero (p_i times calculated population)
1622 cm^{-1}

B: 60 ps (pi times calculated population)
1619.5 cm^{-1}

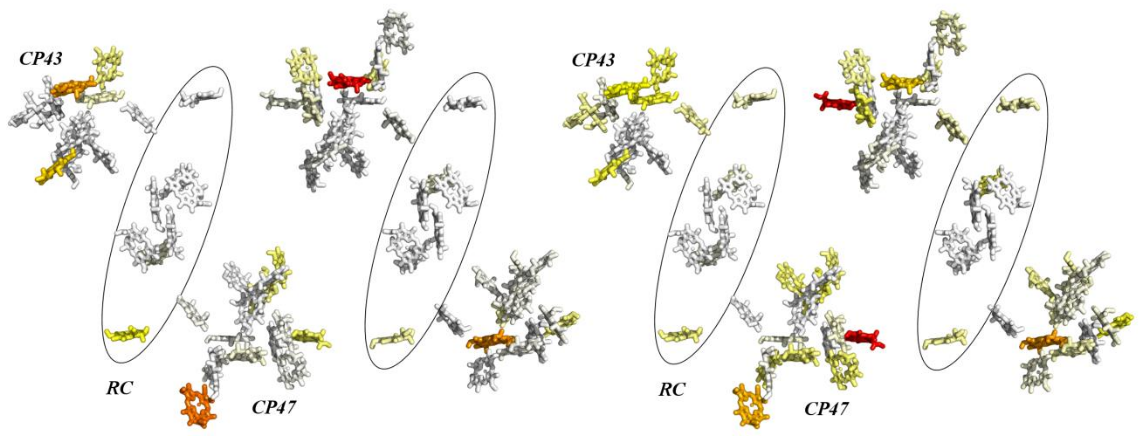

Supplementary Figure 5. Combining experimental dichroic amplitudes at 1622-1619.5 cm^{-1} and calculated populations. Both distributions are the multiplication of calculated population with p_i values, e.g. $\Delta_i(t)$ = Ni(t) * pi. The left figure is the same as presented in the main manuscript in Figure 2B, representing the initial populations in the local basis. The right figure is the corresponding distribution from the calculated populations at 60 ps (see Supplementary Figure 3; left bottom figure for time traces of populations; pumping b- direction) multiplied with the pi values obtained from the 61.5 ps decay spectra (Supplementary Table 3) at the frequency of 1619.5 cm^{-1}. The populations at 60 ps are re-scaled relative to the maximal population

PUMP-B

3ps

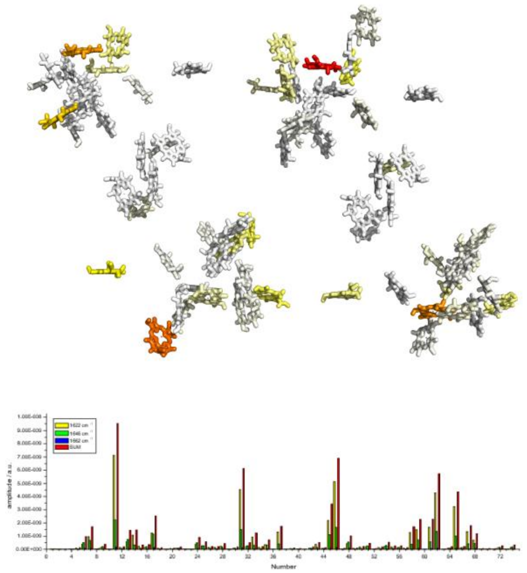

61.5ps

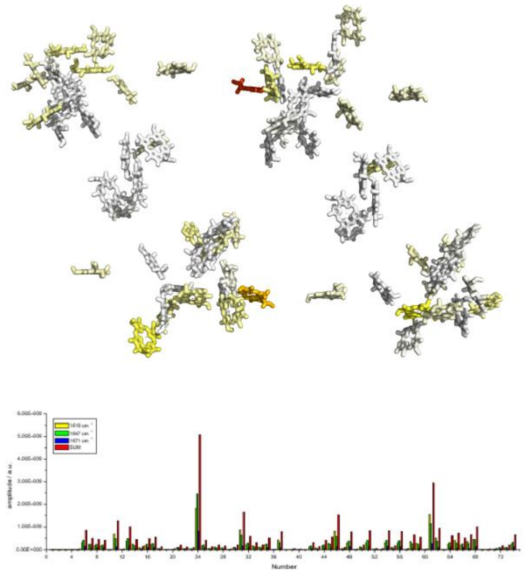

PUMP-C

3ps

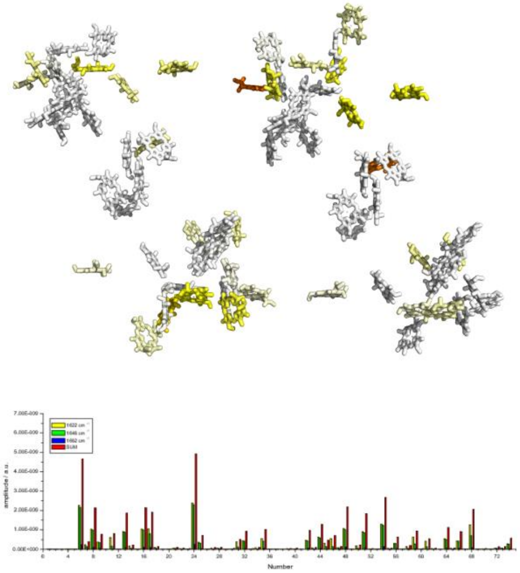

61.5ps

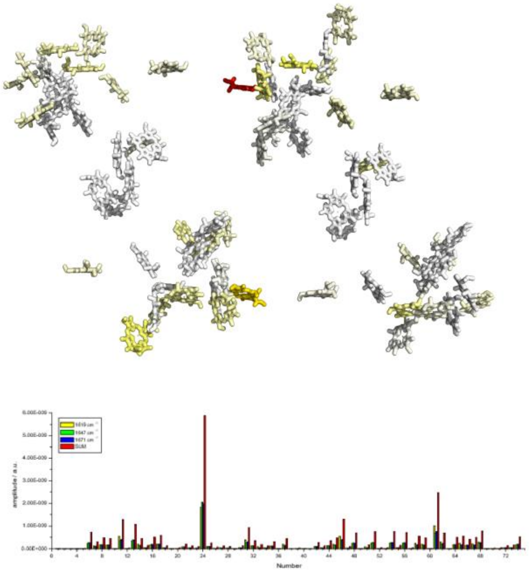

Supplementary Figure 6. Summation of all excited state keto contributions for the 3 ps and 60 ps decay spectra with pumping in the B- (top) or C- direction (bottom). The relative amplitudes are shown as in Supplementary Fig 3 and Fig 2B of the main manuscript as $\Delta_i(t) =$ Ni(t) * pi. The values are scaled to the absorption vector ABC (Supplementary Table 2 for the 3 ps measurements, Supplementary Table 3 for the 61.5 ps measurements) and summed for the molecular presentation. The bar graphs show the individual contributions for each pigment for each contributing band, where the length of the total (red bars) correspond to the coloring on the molecular presentation. The pigment numbering and color coding are the same as in the main text Figure 3.

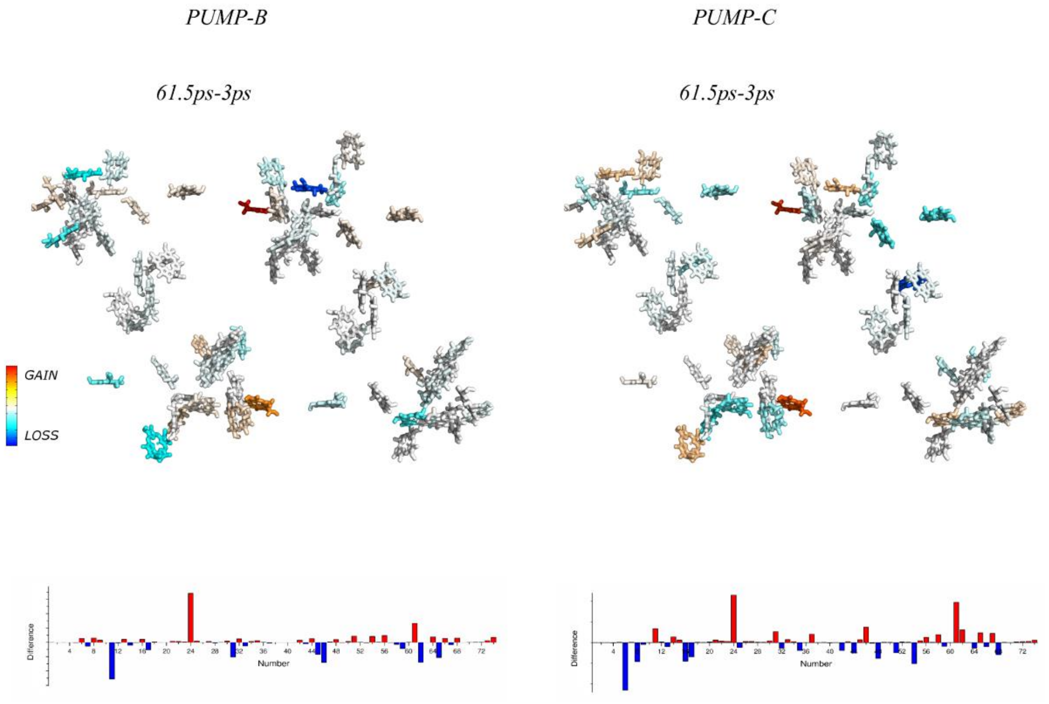

Supplementary Figure 7. The same information presented in Supplementary Figure 6, but as differences between the 61.5 ps and the 3 ps results in order to show relative loss and gain of the exciton populations. The results can be summed for the following domains: Domain 1 (CP47-ChlzD2) Pumping B: + 1.27 (gain), Pumping C: +1.05 (gain). Domain 2 (CP43-ChlzD1) Pumping B: -1.77 (loss), Pumping C: +0.55 (gain). Domain 3 (D1D2 minus Chlz) Pumping B: +0.33 (gain), Pumping C: -1.32 (loss). Note that these values (pi times population) indicate net transfer from CP43 to CP47 but with c- excitation has high initial photoselection of pheophytin (but not in b- direction) which dominates the loss in domain 3. Note also that with pumping the c- direction the calculated population is significantly smaller, about 47% that of the population with pumping the b- direction (see also Supplementary Fig 6). The pigment numbering and color coding are the same as in the main text Figure 3.

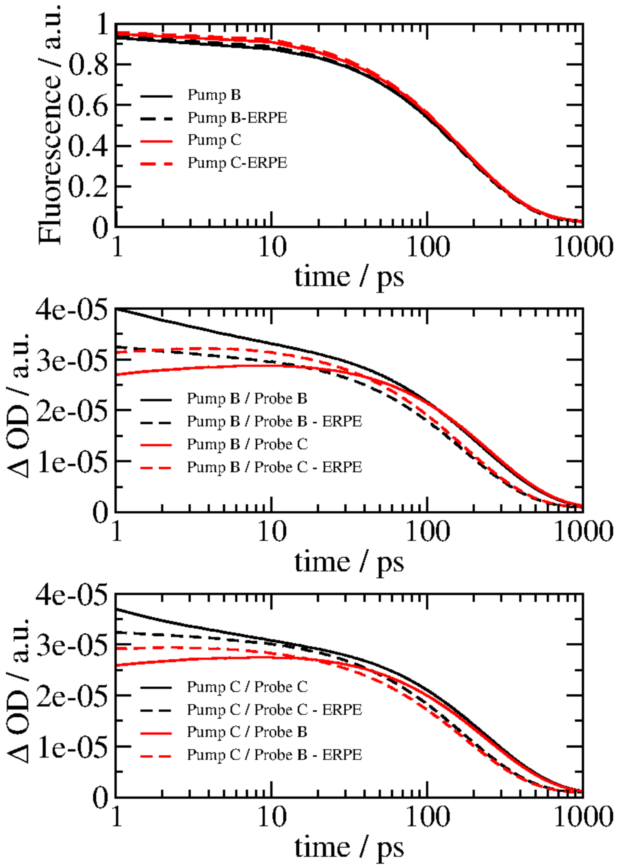

Supplementary Figure 8 Upper part: Decay of isotropic fluorescence for different polarization directions of the excitation pulse (black lines: polarization along the B-axis, red lines: polarization along the C-axis) and in two different models (solid: original structure based model, dashed: fictitious ERPE type model). Middle and Bottom Parts: VIS Pump / IR probe signal calculated for different polarizations of the pump- and probe pulse (as given in the legends) and in two different models (as in upper part).

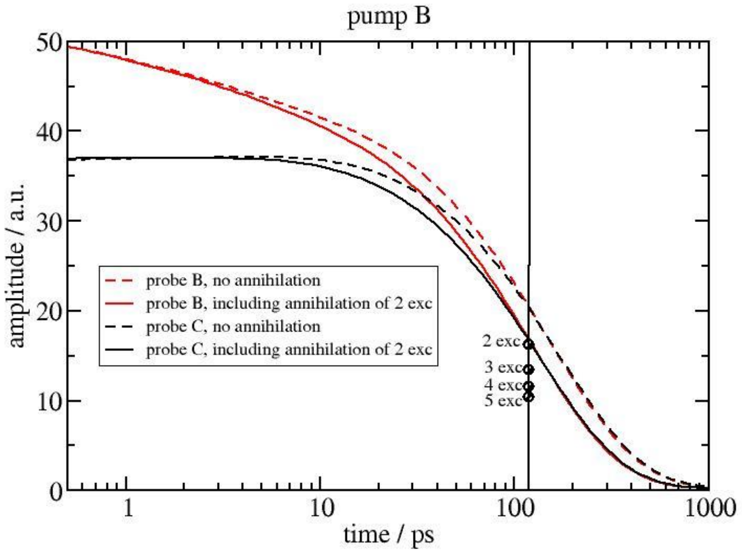

Supplementary Figure 9 VIS Pump / IR probe signal calculated with (solid lines) and without (dashed lines) including exciton-exciton annihilation in the 2 excitation manifold for pump pulse polarization in B-direction and probe pulse polarized in B (red lines) and in C (black lines) directions. For comparison the annihilation free curves have been upscaled. The site energies of Shibata et al.[12] were used in the calculations. The circles at 110 ps denote the amplitude of the signal expected from a simple statistical model, explained in the text, if exciton-exciton annihilation with the 2,3,4 and 5 exciton manifold are included.

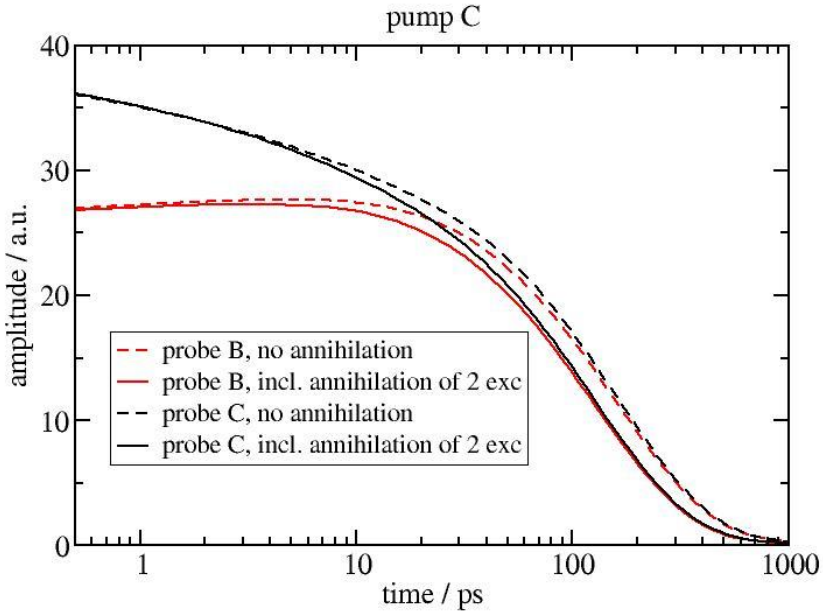

Supplementary Fig. 10 Same pump-probe signals as in Supplementary Fig. 9, but for pump-pulse polarized parallel to the crystal c-axis.

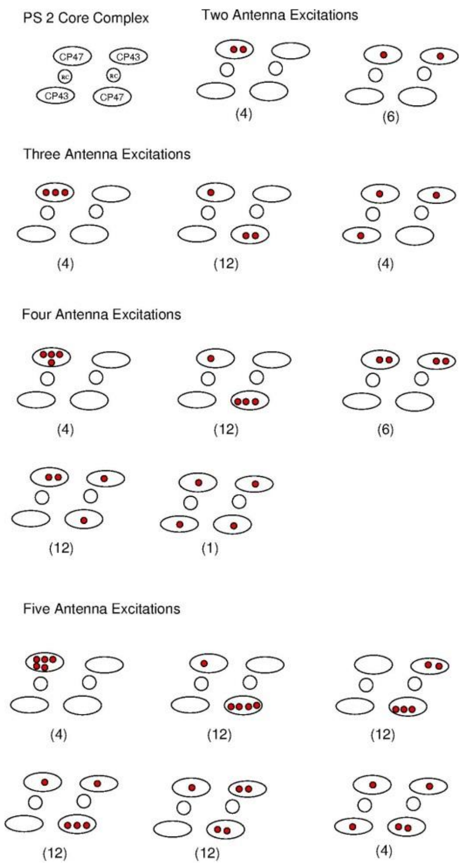

Supplementary Figure 11: Illustration of different realizations of multiple antenna excitations states. The structure of the PS 2 core complex dimer (shown in the upper left corner) contains two reaction centers and one CP43 and CP47 antenna subunit per RC. A single red dot in a subunit represents a single excitation present in one of the exciton domains belonging to this subunit. Multiple red dots in the same subunit, describe multiple excitations present in a subunit are likely to experience exciton-exciton annihilation, as explained in the text. The numbers below the different states denote the statistical weight, which describes the number of microscopic realizations of that state. These statistical weights and the above states are used in the text to estimate the influence of exciton-exciton annihilation on the amplitude of the pump-probe signal at large delay times.

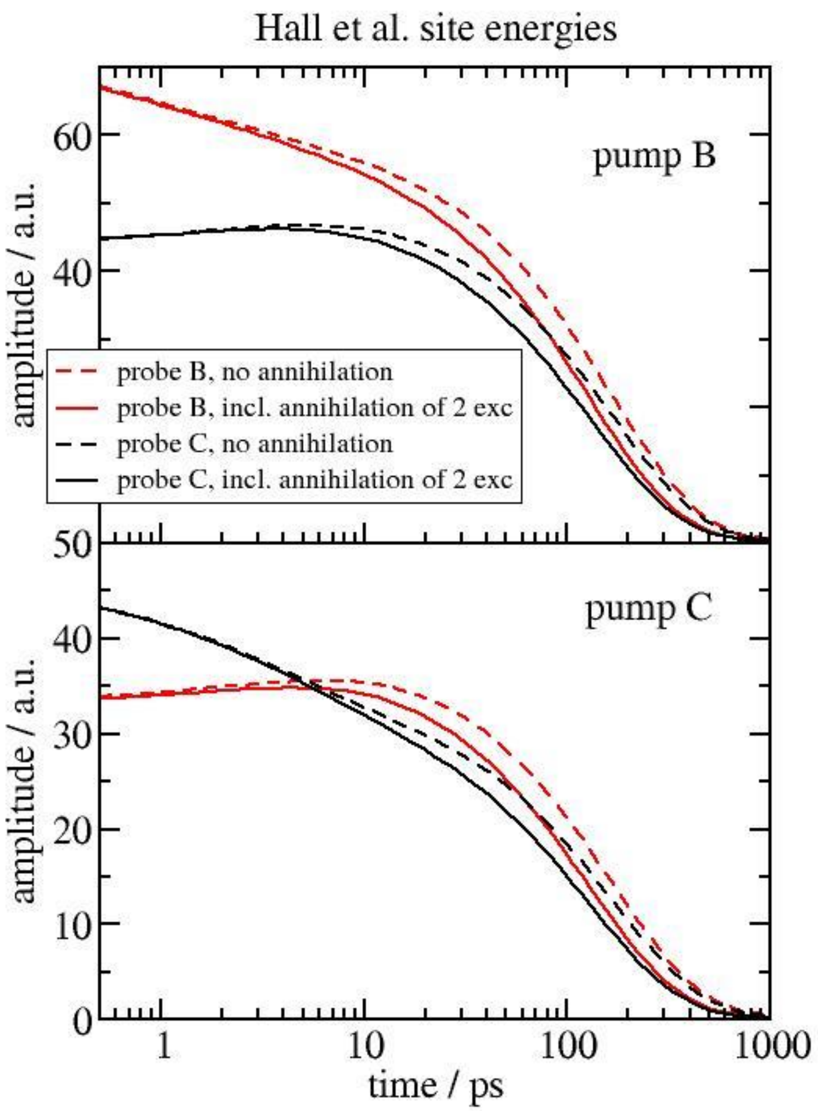

Supplementary Figure 12 Same calculations as in Supplementary Figures 9 and 10, except for a replacement of the site energies of CP47 by recent values of Hall et al.[14]. These values (in corresponding wavelengths in nm) are: Chl 11: 687.5, Chl 12: 665, Chl 13: 672, Chl 14: 672, Chl 15: 662, Chl 16: 676, Chl 17: 676, Chl 21: 668, Chl 22: 669, Chl 23: 666.5, Chl 24: 683, Chl 25: 663, Chl26: 669, Chl 27: 679, Chl 28: 670, Chl 29: 689.5.

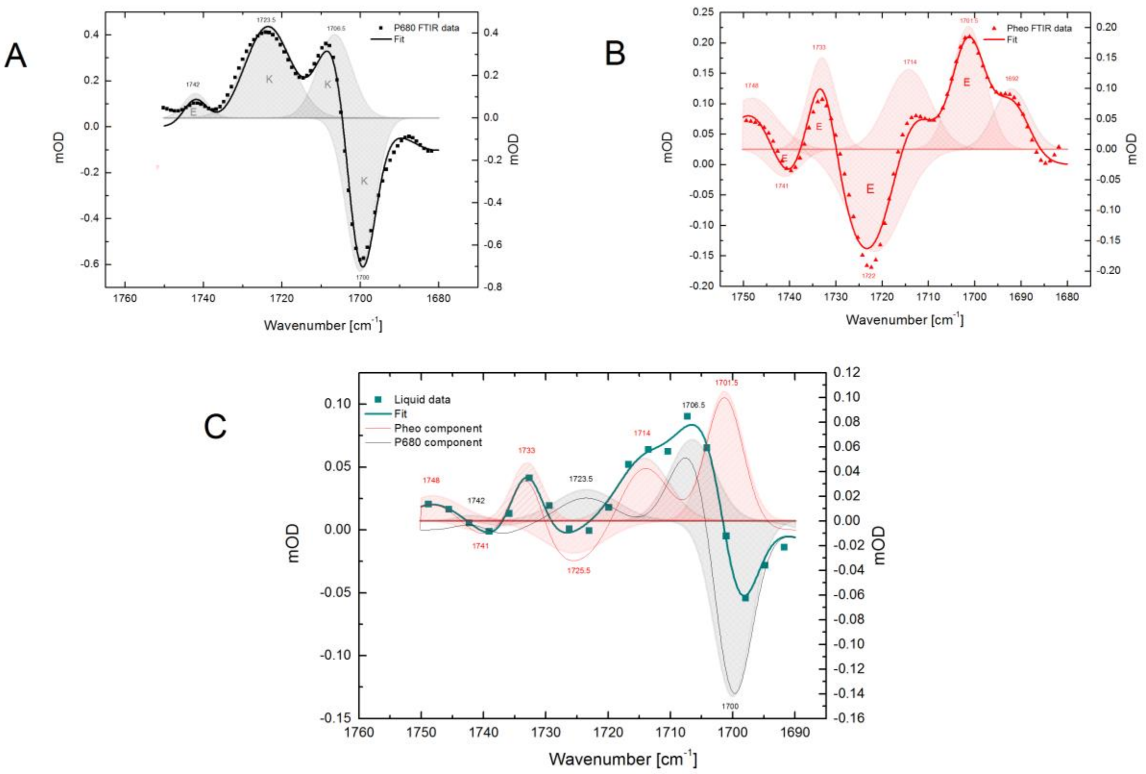

Supplementary Figure 13 A. Difference FTIR spectrum of P680$^+$/P680 from (Ref.9) and its fit using set of Gaussian bands. B. Difference FTIR spectrum of Pheo$^-$/Pheo from (Ref. 10) and its fit using set of Gaussian bands. C. 1 ns TRIR difference spectrum of isotropic PSII sample and its fit using set of Gaussian bands.

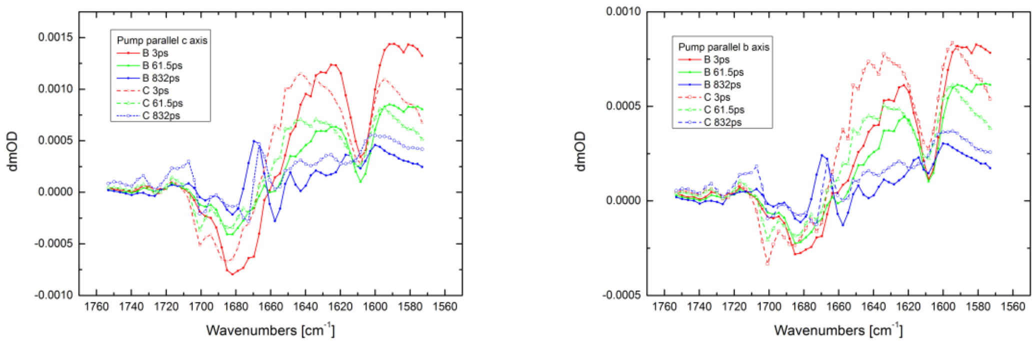

Supplementary Figure 14. Global Analysis results of the PSII crystal TRIR. The results for the case when pump is parallel to c axis are presented left, when pump is parallel b axis – right. Solid line spectra correspond to probe parallel to b axis, dashed – parallel to c axis in both panels. For each case there are three spectra corresponding to 3 ps, 61.5 ps and 832 ps time constants.

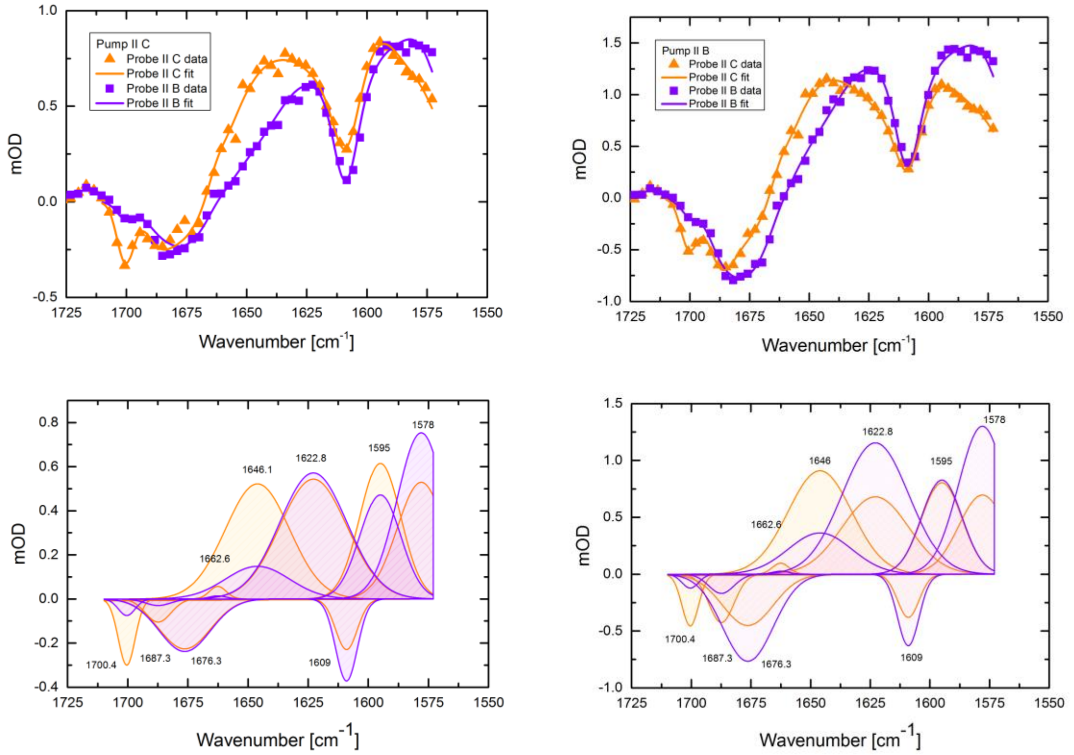

Supplementary Figure 15. Results of band fitting the 3 ps spectra. Left pump is parallel to the c axis and right – to the b axis. In the upper panel the points represent GA 3 ps spectrum and solid line – band fitting result for the two probe polarizations: parallel to the c axis (orange), parallel to b axis (purple). Lower panel shows bands used to fit the spectrum. There are two versions of each band corresponding to the two spectra in the upper panel as indicated by the color of the band.

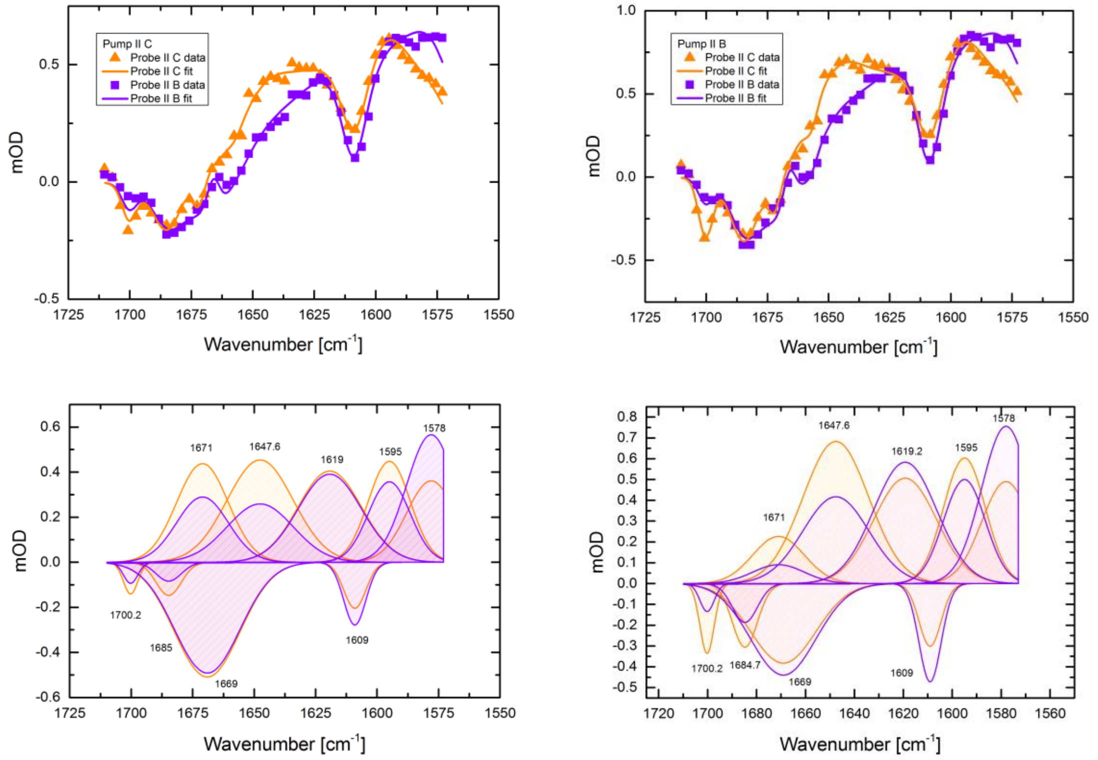

Supplementary Figure 16. Results of band fitting the 61.5 ps spectra. Left pump is parallel to the c axis and right – to the b axis. In the upper panel the points represent GA 3 ps spectrum and solid line – band fitting result for the two probe polarizations: parallel to the c axis (orange), parallel to b axis (purple). Lower panel shows bands used to fit the spectrum. There are two versions of each band corresponding to the two spectra in the upper panel as indicated by the color of the band.

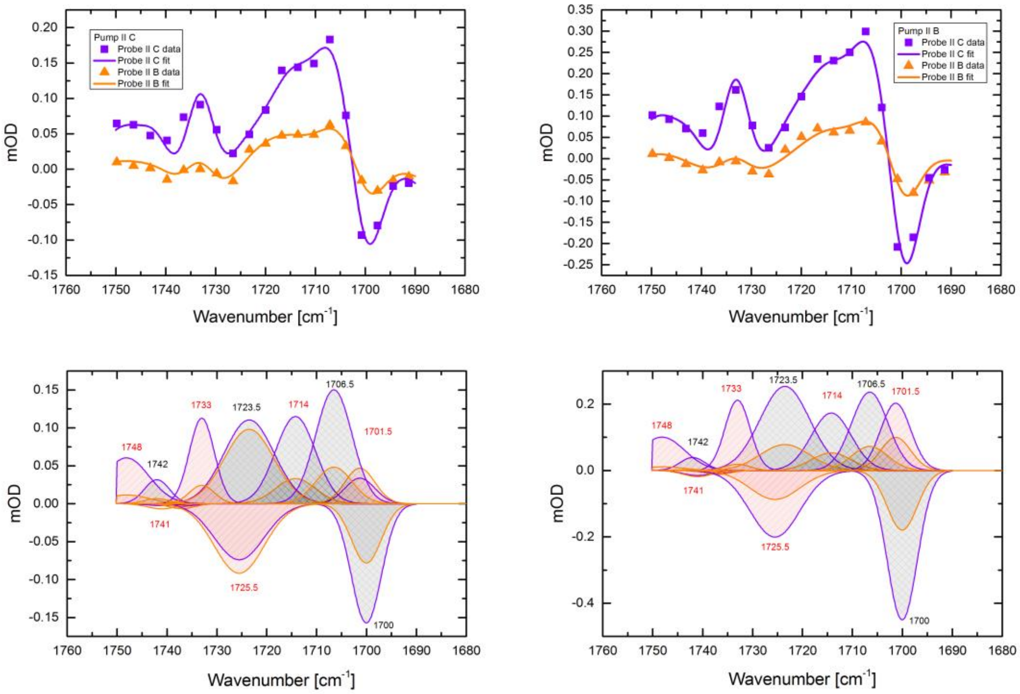

Supplementary Figure 17. Results of band fitting the 832 ps spectra. Left pump is parallel to the c axis and right – to the b axis. In the upper panel the points represent GA 3 ps spectrum and solid line – band fitting result for the two probe polarizations: parallel to the c axis (orange), parallel to b axis (purple). Lower panel shows bands used to fit the spectrum. There are two versions of each band corresponding to the two spectra in the upper panel as indicated by the color of the band.

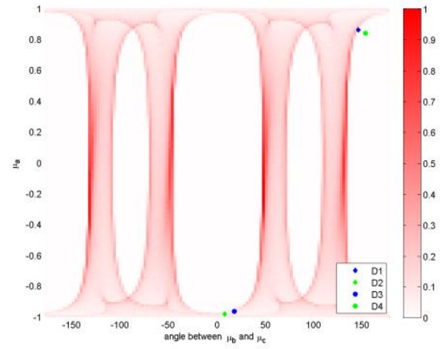

Supplementary Figure 18. Comparison of TDM orientation from DFT calculations and all possible dipole orientations that could produce experimental results for the 1700 cm^{-1} band. This band was assigned to 13-1-keto C=O neutral vibration. D1 and D2 correspond to keto modes of the PD1, D3 and D4 – of PD2.

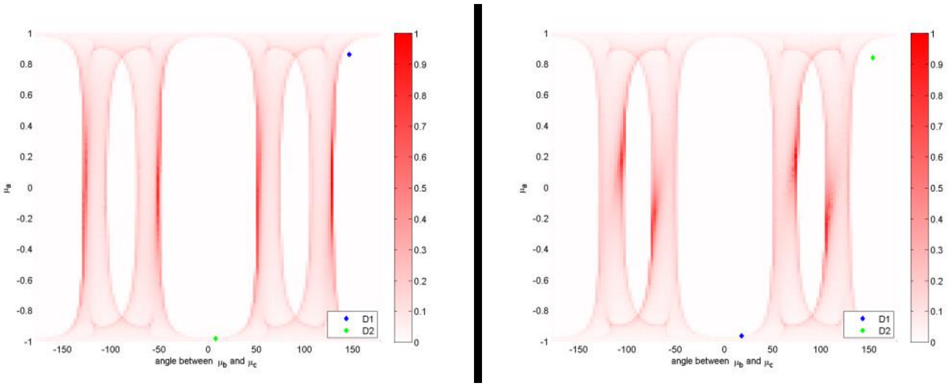

Supplementary Figure 19. Comparison of TDM orientation from DFT calculations and all possible dipole orientations that could produce experimental results for the 1706 cm^{-1} band (left) and 1723 cm^{-1} band (right) . 1706 cm^{-1} band was assigned to PD1 13-1-keto C=O cation vibration. 1723 cm^{-1} band was assigned to PD1 13-1-keto C=O cation vibration.

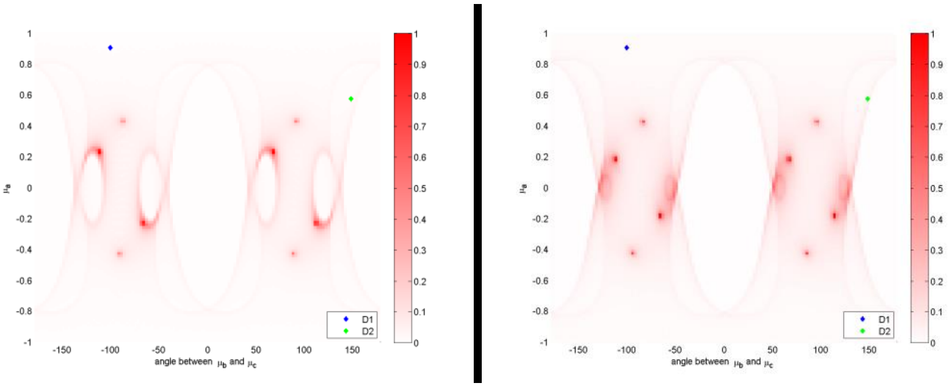

Supplementary Figure 20 . Comparison of TDM orientation from DFT calculations and all possible dipole orientations that could produce experimental results for the 1701 cm^{-1} band (left) and 1725 cm^{-1} band (right) . 1701 cm^{-1} band was assigned to Pheo 13-2-ester C=O anion vibration. 1725 cm^{-1} band was assigned to Pheo 13-2-ester C=O neutral vibration.

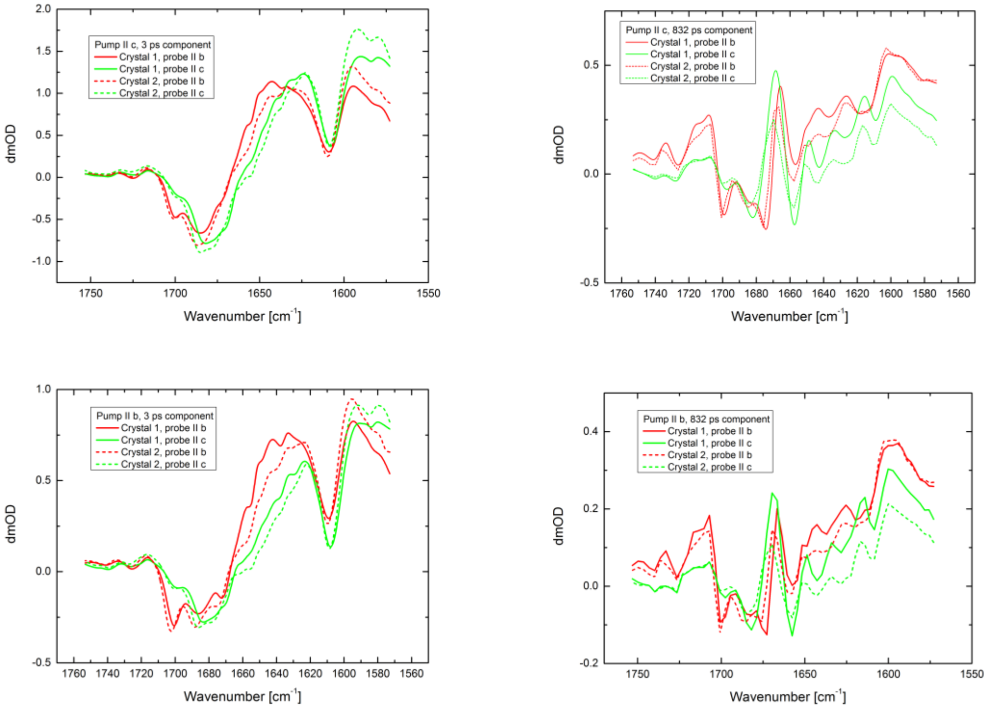

Supplementary Figure 21 Comparison of GA spectra for two different crystals. Solid line indicates spectra of the crystal used in the analysis and dashed line – of another crystal. The spectra corresponding to 3 ps time constant are shown on the left and the ones corresponding to 832 ps constant – on the right.

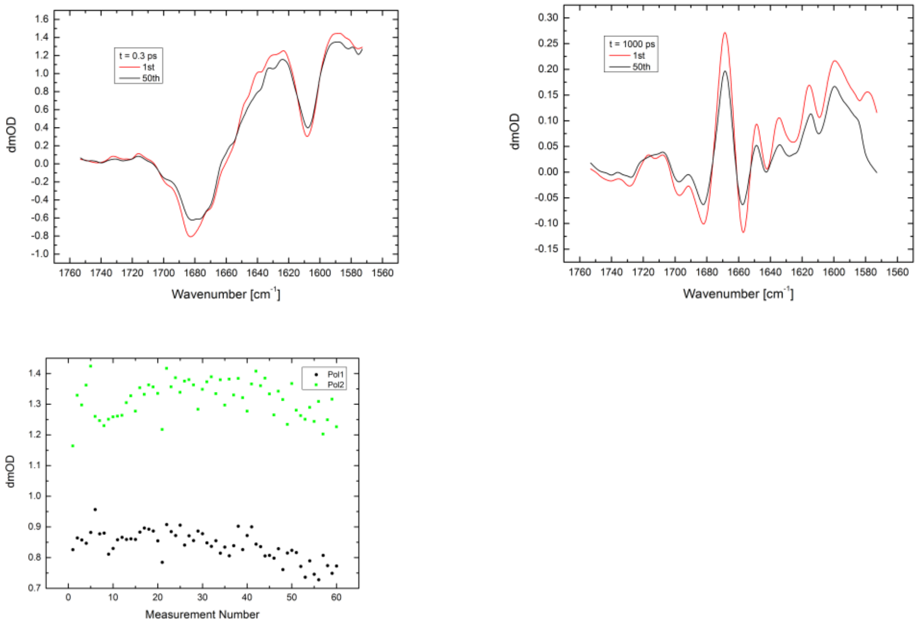

Supplementary Figure 22 Assessment of damage to the crystal during the measurement. The two upper panels show spectra of measured on the same crystal at two different delays at the beginning and towards the end of the measurement – the first and 50th measurements. Average of ten consecutive measurements (1st to 10th and 50th to 60th) is presented in upper right panel due to low signal. Lower panel shows the amplitude of signal at particular wavelength as a number of measurements.

Supplementary Table 1.
First column shows the numbering of the molecules as used in this paper. Next three columns show corresponding label used by other authors. The TD-DFT section of the table show corresponding site energies and orientation of the transition dipole obtained using TD-DFT calculations. The last six columns show orientation of the keto and ester groups of the molecules obtained from X-ray crystal structure and DFT calculations. The site energies were taken from Shibata et al (Ref 12) as indicated in the table

Exc. Code	Loll et al	Shen	Shibata et al	TD-DFT				Keto			Ester		
	2AXT pdb	3ARC. pdb		site energy									
					a	b	c	a	b	c	a	b	c
1	558	604	PD1	664	0.77	-0.37	0.52	2.58	-1.27	0.86	-2.17	-0.93	-1.85
2	354	605	PD2	668.5	-0.99	0.02	0.15	-2.89	0.78	0.25	2.51	1.21	1.13
3	559	606	AccD1	682	-1.00	0.04	0.06	-2.93	0.29	-0.56	2.33	1.82	-0.52
4	560	607	AccD2	667	0.78	-0.43	0.46	2.73	-0.73	1.02	-1.04	0.47	-2.77
5	561	608	PheD1	667.5	0.63	0.66	-0.40	2.43	1.48	-0.96	-0.74	-1.11	2.69
6	562	609	PheD2	675	-0.01	0.23	-0.97	-0.73	0.96	-2.75	-1.42	-1.80	1.93
7	563	610	ChlZD1	666	-0.25	-0.82	0.52	-0.33	-2.11	2.10	2.44	1.30	-1.17
8	355	611	ChlZD2	669.5	-0.38	-0.10	0.92	-1.66	-0.72	2.40	-0.73	1.20	-2.65
9	511	612	Chl11	681	-0.63	0.10	0.77	-2.32	0.64	1.79	1.64	1.70	-1.85
10	512	613	Chl12	664.5	0.98	0.18	-0.05	2.87	0.62	0.60	-2.54	1.43	0.69
11	513	614	Chl13	675.5	0.03	-1.00	-0.02	0.64	-2.90	0.46	1.74	2.34	-0.71
12	514	615	Chl14	667	-0.77	0.63	0.10	-2.65	1.40	-0.14	1.83	-1.04	-2.14
13	515	616	Chl15	677.5	0.75	0.01	0.66	1.71	0.29	2.45	-1.81	1.93	-1.42
14	516	617	Chl16	669.5	-0.66	-0.73	-0.16	-1.36	-2.58	-0.70	1.90	1.75	-1.53
15	517	618	Chl17	659	0.54	-0.82	-0.18	1.61	-2.52	0.21	0.30	2.68	1.31
16	518	619	Chl21	671	-0.65	-0.15	-0.74	-1.82	0.29	-2.36	0.24	1.56	2.55
17	519	620	Chl22	670.5	-0.37	0.64	0.67	-0.41	1.85	2.33	0.98	-2.83	-0.16
18	520	621	Chl23	656	0.02	-0.61	-0.79	0.40	-2.30	-1.89	1.91	1.47	1.79
19	521	622	Chl24	683.5	0.91	0.37	0.17	2.94	0.61	-0.04	-1.51	-2.58	0.26
20	522	623	Chl25	663.5	-0.69	0.71	0.13	-2.55	1.57	0.23	1.07	-1.78	-2.17
21	523	624	Chl26	669.5	0.68	-0.59	-0.43	1.96	-1.28	-1.88	-2.87	0.82	-0.35
22	524	625	Chl27	675	0.95	0.16	-0.27	2.62	0.13	-1.46	-1.87	-2.28	0.57
23	525	626	Chl28	670.5	0.96	0.10	0.26	2.59	0.22	1.49	-2.61	1.44	0.30
24	526	627	Chl29	689.5	0.02	0.33	-0.94	0.55	0.40	-2.92	1.83	-0.32	2.36
25	491	628	Chl33	669	-0.38	0.54	0.75	-1.80	1.27	2.04	-0.18	-0.19	-2.99
26	492	629	Chl34	666.5	0.68	-0.72	0.14	2.26	-1.71	1.00	-0.13	2.90	-0.77
27	493	630	Chl35	669	-0.83	-0.42	-0.38	-2.18	-1.88	-0.84	2.98	-0.09	-0.37
28	494	631	Chl37	677	-0.67	0.58	0.46	-2.48	1.16	1.23	0.52	-1.19	-2.70
29	495	632	Chl41	669	0.84	0.53	0.04	2.70	1.19	-0.51	-0.92	-2.85	0.24
30	496	633	Chl42	669	0.86	-0.42	-0.29	2.14	-1.54	-1.42	-2.71	-0.80	1.01
31	497	634	Chl43	676.5	0.13	0.94	0.33	-0.35	2.76	1.11	-1.42	-1.47	-2.19
32	498	635	Chl44	671.5	-0.60	-0.04	-0.80	-1.87	0.64	-2.26	-0.15	0.55	2.94
33	499	636	Chl45	679.5	0.77	-0.64	-0.06	2.44	-1.66	0.54	-0.50	2.95	0.20
34	500	637	Chl46	658	-0.61	0.77	-0.15	-1.28	2.56	-0.90	2.69	-0.58	1.19
35	501	638	Chl47	666	-0.21	0.40	-0.89	-0.45	1.85	-2.32	-1.03	0.03	2.82
36	502	639	Chl48	670.5	-0.90	-0.06	-0.42	-2.73	-0.85	-0.92	2.67	-1.36	-0.12
37	503	640	Chl49	662	-0.08	0.99	0.09	0.53	2.94	0.28	0.99	-2.37	1.56
38	5558	604	PD1	664	-1.00	-0.05	0.07	-2.94	0.60	0.08	2.34	1.23	1.43
39	5354	605	PD2	668.5	0.74	-0.46	0.48	2.51	-1.47	0.73	-2.17	-0.61	-1.98
40	5559	606	AccD1	682	0.81	-0.39	0.44	2.77	-0.58	0.99	-1.08	0.52	-2.75

Supplementary Table 1 (continued).

Exc. Code	Loll et al	Shen	Shibata et al	TD-DFT				Keto			Ester		
	2AXT pdb	3ARC. pdb		site energy									
41	5560	607	AccD2	667	-0.99	0.02	0.10	-2.95	0.30	-0.43	2.30	1.87	-0.48
42	5561	608	PheD1	667.5	-0.11	0.30	-0.95	-1.06	1.06	-2.60	-1.01	-2.14	1.85
43	5562	609	PheD2	675	0.54	0.75	-0.39	2.20	1.76	-1.04	-0.34	-1.45	2.61
44	5563	610	ChlZD1	666	-0.31	-0.22	0.93	-1.41	-1.11	2.41	-1.03	1.29	-2.51
45	5355	611	ChlZD2	669.5	-0.15	-0.88	0.45	0.01	-2.34	1.88	2.23	1.59	-1.23
46	5511	612	Chl11	681	0.20	-0.91	0.37	1.31	-2.55	0.90	0.07	1.47	-2.61
47	5512	613	Chl12	664.5	-0.72	0.30	-0.62	-2.43	0.18	-1.75	2.25	-1.98	0.11
48	5513	614	Chl13	675.5	-0.34	0.40	0.85	-1.69	0.92	2.30	-0.32	0.31	-2.97
49	5514	615	Chl14	667	0.80	-0.58	-0.17	2.71	-1.28	0.01	-0.87	2.84	-0.40
50	5515	616	Chl15	677.5	-0.92	-0.32	-0.21	-2.44	-1.65	-0.54	2.78	-0.11	-1.12
51	5516	617	Chl16	669.5	0.37	0.20	0.91	0.57	1.15	2.71	-0.26	1.28	-2.70
52	5517	618	Chl17	659	-0.60	0.47	0.64	-2.27	0.81	1.79	0.08	-0.81	-2.89
53	5518	619	Chl21	671	0.83	0.48	0.28	2.69	1.33	0.12	-0.85	-2.72	-0.94
54	5519	620	Chl22	670.5	0.64	-0.13	-0.76	1.26	-0.53	-2.67	-1.97	-1.57	1.63
55	5520	621	Chl23	656	0.14	0.92	0.36	-0.25	2.64	1.41	-1.88	-1.49	-1.80
56	5521	622	Chl24	683.5	-0.70	0.01	-0.71	-2.18	0.75	-1.92	0.23	0.28	2.98
57	5522	623	Chl25	663.5	0.75	-0.61	-0.26	2.52	-1.62	-0.10	-0.49	2.90	0.60
58	5523	624	Chl26	669.5	-0.56	0.82	0.10	-1.17	2.75	-0.21	2.79	-0.95	0.57
59	5524	625	Chl27	675	-0.60	0.49	-0.63	-1.44	2.08	-1.61	0.51	-0.25	2.95
60	5525	626	Chl28	670.5	-0.87	0.06	-0.48	-2.75	-0.49	-1.10	2.47	-1.70	0.04
61	5526	627	Chl29	689.5	0.53	0.70	-0.49	1.03	2.55	-1.19	-2.70	-1.31	-0.10
62	5491	628	Chl33	669	0.16	-0.98	-0.13	0.99	-2.83	0.17	1.44	2.61	-0.35
63	5492	629	Chl34	666.5	-0.86	0.38	0.33	-2.89	0.55	0.59	1.46	-0.48	-2.58
64	5493	630	Chl35	669	0.71	0.22	0.67	1.53	0.74	2.48	-2.30	1.32	-1.40
65	5494	631	Chl37	677	0.54	-0.84	-0.09	1.87	-2.31	0.42	0.40	2.96	0.23
66	5495	632	Chl41	669	-0.53	0.05	-0.84	-1.58	0.89	-2.39	-0.31	0.56	2.93
67	5496	633	Chl42	669	-0.72	0.69	-0.10	-1.62	2.53	0.02	1.49	-1.44	2.17
68	5497	634	Chl43	676.5	0.05	-0.59	-0.80	0.71	-2.12	-2.00	1.68	1.99	1.49
69	5498	635	Chl44	671.5	0.86	0.50	0.15	2.81	1.05	-0.14	-1.09	-2.79	0.19
70	5499	636	Chl45	679.5	-0.82	0.54	0.18	-2.81	0.97	0.38	1.32	-1.46	-2.26
71	5500	637	Chl46	658	0.84	-0.37	-0.41	2.33	-0.61	-1.79	-2.96	0.07	-0.52
72	5501	638	Chl47	666	0.72	0.55	-0.43	2.07	1.13	-1.85	-0.46	-2.78	1.03
73	5502	639	Chl48	670.5	0.92	0.10	0.39	2.37	0.24	1.82	-2.61	1.49	-0.11
74	5503	640	Chl49	662	0.36	-0.49	-0.80	0.44	-1.19	-2.72	-2.33	-0.12	1.89

Supplementary Table 2. The results of band fitting for 3 ps spectrum.
First column shows centre frequency (in cm^{-1}) of the band used in fitting of the spectrum. Second column – its direction. Following columns show resulting amplitudes for all possible pump and probe polarizations. D.R. stands for dichroic ratio defined as: $I_{\| C}/ I_{\| B}$

		Pump II B			Pump II C		
Center Wn	Direction	Probe Pol II C	Probe Pol II B	D.R.	Probe Pol II C	Probe Pol II B	D.R.
1578	+	6.96E-04	1.30E-03	0.53	5.29E-04	7.53E-04	0.70
1595	+	8.04E-04	8.27E-04	0.97	6.15E-04	4.72E-04	1.30
1609	-	3.80E-04	6.29E-04	0.60	2.29E-04	3.72E-04	0.62
1622.8	+	6.80E-04	1.15E-03	0.59	5.43E-04	5.71E-04	0.95
1646.1	+	9.10E-04	3.62E-04	2.51	5.22E-04	1.48E-04	3.52
1662.6	+	9.68E-05	2.61E-05	3.71	5.70E-05	1.42E-05	4.01
1676.3	-	4.51E-04	7.66E-04	0.59	2.26E-04	2.38E-04	0.95
1687.3	-	4.25E-04	1.69E-04	2.51	1.05E-04	2.98E-05	3.52
1700.4	-	4.55E-04	1.23E-04	3.71	3.00E-04	7.47E-05	4.01

Supplementary Table 3. Results of the band fitting the 61.5 ps spectrum

First column shows centre frequency (in cm^{-1}) of the band used in fitting of the spectrum. Second column – its direction. Following columns show resulting amplitudes for all possible pump and probe polarizations. D.R. stands for dichroic ratio defined as: $I_{\parallel C}/ I_{\parallel B}$

		Pump II B			Pump II C		
Center Wn	Direction	Probe Pol II C	Probe Pol II B	D.R.	Probe Pol II C	Probe Pol II B	D.R.
1578	+	4.90E-04	7.56E-04	0.65	3.61E-04	5.66E-04	0.64
1595	+	6.03E-04	5.00E-04	1.21	4.48E-04	3.57E-04	1.25
1609	-	3.01E-04	4.72E-04	0.64	2.04E-04	2.78E-04	0.73
1619.2	+	5.06E-04	5.83E-04	0.87	4.05E-04	3.91E-04	1.04
1647.6	+	6.83E-04	4.17E-04	1.64	4.54E-04	2.59E-04	1.75
1671	+	2.27E-04	9.05E-05	2.50	4.38E-04	2.89E-04	1.52
1669.1	-	3.82E-04	4.40E-04	0.87	5.09E-04	4.92E-04	1.03
1684.7	-	3.06E-04	1.87E-04	1.64	1.47E-04	8.37E-05	1.76
1700.2	-	3.36E-04	1.34E-04	2.51	1.41E-04	9.32E-05	1.51
1657	-	1.07E-04	6.11E-05	1.75	2.19E-05	1.28E-12	Inf
1666	+	1.26E-11	2.15E-04	0.00	7.51E-12	1.28E-04	0.00
1672	-	1.80E-04	1.06E-11	Inf	1.25E-04	7.32E-12	Inf

Supplementary Table 4. Results of the band fitting of the 832 ps spectrum

First column shows centre frequency (in cm^{-1}) of the band used in fitting of the spectrum. Second column – its direction. Next possible assignments of the band is indicated. See SI text for more details. Following columns show resulting amplitudes for all possible pump and probe polarizations.

					Pump II B		Pump II C	
Center Wn	Direction	assign.			Probe Pol II C	Probe Pol II B	Probe Pol II C	Probe Pol II B
1701.3	+	pheo	ester		2.03E-04	9.99E-05	3.37E-05	4.68E-05
1714.2	+	pheo	?		1.73E-04	5.34E-05	1.15E-04	3.31E-05
1725.5	-	pheo	ester		-2.01E-04	-8.70E-05	-7.39E-05	-9.17E-05
1733	+	pheo	ester		2.12E-04	1.89E-05	1.13E-04	2.42E-05
1741	-	pheo	ester		-1.49E-05	-1.84E-05	-2.47E-06	-6.82E-06
1700	-	p680	keto	PD1, PD2	-4.50E-04	-1.79E-04	-1.57E-04	-7.82E-05
1706.5	+	p680	keto	PD2	2.36E-04	7.27E-05	1.50E-04	4.82E-05
1723.5	+	p680	keto	PD1	2.54E-04	7.81E-05	1.10E-04	9.80E-05
1737	-	p680	ester	PD1, PD2	-9.44E-06	-1.16E-05	-3.30E-06	-6.56E-06
1742	+	p680	ester	PD1, PD2	3.91E-05	3.01E-06	3.17E-05	6.79E-06

Supplementary Methods

Experimental probes of ultrafast structural dynamics in proteins gain important structural information when conducted in crystalline form [1], in combination with real-space analysis possible from recent high resolution X-ray structures. Using previously reported and characterized instrumentation that allows femtosecond time resolved pump-probe mid-infrared spectroscopy in diffraction-limited spots [2] we have performed polarization-resolved measurements in transmission mode of small oriented single crystals of PSII core complexes of *Synechococcus elongatus* with known index [3,4]. The X-ray structure of *S. elongatus* PSII core complexes has initially been reported in the orthorhombic space group $P2_12_12_1$ at 3.0 Å resolution, from which the coordinates of 74 chlorins in the dimer structure have been analysed [3]. Polarized infrared crystallography of orthorhombic space groups, which are classified as biaxial, allows explicit analysis of the molecular transition dipole directions directly in the coordinate frame [1]. Generally, two mutually orthogonal polarized waves satisfy Maxwell's general wave equation in crystals, except for those having cubic space groups [5]. Because of symmetry considerations, the directions of the principal dielectric axes ($\varepsilon_x \neq \varepsilon_y \neq \varepsilon_z$) of orthorhombic crystals correspond to the crystallographic symmetry axes (a,b,c) and are wavelength independent [5]. For oriented orthorhombic crystals with a *k* vector in the direction of the crystallographic a- axis the two allowed polarized waves have the *E* field in the directions that correspond to the crystallographic b- and c- axes [5]. For a molecule having a transition dipole moment (TDM) μ_1 the linear response for polarized waves with the *E* field in the e_1 and e_2 directions are then proportional to $(e_1.\mu_1)^2$ and $(e_2.\mu_1)^2$, respectively (Supplementary Figs 1, 2). Since the quantities $(e_1.\mu_1)^2$ and $(e_2.\mu_1)^2$ are invariant under each two-fold operation, all n copies of molecules present in the asymmetric unit are summed in order to find the linear response for polarized waves for the unit cell in orthorhombic crystals. Although non-crystallographic symmetry is not perfect for *S. elongatus* PSII cores, n is taken to be 2 for the dimer structure in the asymmetric unit in the 2AXT pdb structure [3]. These properties are exploited in the present study in order to extract structural information for ultrafast infrared spectroscopy measurements of PSII cores.

It has been pointed out that time resolved fluorescence measurements of core complexes of PSII from *S. elongatus* could be modeled using either the ERPE model or a transfer to the trap limited model [6]. Rather, a preference for the latter model was developed from theoretical arguments based on the long distances seen between CP43 and CP47 antenna pigments and the RC in the crystal structure. By comparing visible pump/infrared probe experiments on whole PSII core complexes and on the isolated subunit, slow transfer times between the CP43/CP47 subunits and the reaction center were inferred [7]. Here, we will present the first direct experimental proof of the validity of the transfer-to-trap limited model from experiments on core-complexes only.

Ultrafast measurements of exciton dynamics are performed in order to retrieve explicit structurally sensitive parameters which may be compared to existing theory and modeling. It is shown that in the mid-infrared spectral region the resulting time dependent signals can be analyzed in a local site basis; four individual experimental conditions are thus considered: with the optical pump having either b- or c- polarization and probing either in b- or c- direction.

Laboratory orientation of orthorhombic PSII core crystals

Theoretical modeling of the exciton processes in PSII requires precise knowledge of atomic coordinates for each molecule in the core complex. The *Synechococcus elongatus* X-ray structure was compared with the more recently published 1.9 Å resolution structure of *Thermosynechococcus vulcanus* PSII core complexes [8]. The structures were found to be highly homologous with very small RMS differences in the orientations (0.78 Å RMS differences for Cα carbons [8]). The orientation and coordinate differences were also very small for most chlorine chromophores, except Chlorophyll '11' (a peripheral CP47 pigment) which was rotated by in-plane ~90° in the case of *T. vulcanus* structure [3,4,8]. We used the pigment orientations from the *S elongatus* structure except for Chl 11 which had the orientation from the *T. vulcanus* structure.

For calculations that have c-polarized excitation one of the Chl 29 molecules is initially populated (nr. 24) while the second copy is not and only becomes populated at long delays (nr 61). The calculations show that during the first 100 ps, the direction of the excitation polarization has substantial effect on the energy transfer paths taken as a result of the initial photoselection. However, at delay times longer than 100 ps the difference in population for two excitation polarizations gradually disappears. Moreover, the population of the two monomers in the asymmetric unit also becomes similar and non-crystallographic symmetry is developed at about 200 ps (Supplementary Figure 3). The same results are represented as the differences between 60 ps and 3 ps to show the relative loss and gain in exciton population for both directions of pumping (See Supplementary Fig. 7).

Structural measurement of the charge separated state $P_{680}^{+}/Pheo^{-}$

With a ~ 800 ps time constant, the formation of the charge separated state $P680^{+}/Pheo^{-}$ is observed (Supplementary Figure 17). There are pronounced differences between absorption of the B-polarized and C-polarized probe in the 1690 – 1740 cm^{-1} region. However, as expected, the spectra no longer differ for two excitation polarizations. Furthermore, because at this stage both monomers in the crystallographic asymmetric unit are equally populated, there exists a fully developed non-crystallographic symmetry.

A band-fitting analysis was based on previously published FTIR difference spectra and assignments for $P680^{+}/P680$ [9] and $Pheophytin^{-}/Pheophytin$ [10]. A set of bands were simultaneously fitted to the $P680^{+}/P680$ FTIR, $Pheo^{-}/Pheo$ FTIR, the polarization resolved $P680^{+}/Pheo^{-}$ crystal spectra as well as the $P680^{+}/Pheo^{-}$ spectrum of isotropic sample acquired under identical conditions as crystallographic data (Supplementary Figure 13, 17). A discussion and summary of the assignments is presented below and Supplementary Table 3 presents the fitted experimental amplitudes.

There is generally very good agreement between set of bands needed to fit TRIR and FTIR spectra. Both Chla 13-1-keto C=O keto and Pheo 13-1-keto C=O as well as 13-2-ester C=O bands can be reliably fitted. The corresponding theoretically calculated TDM directions are presented in Supplementary Figure 4 and Supplementary Table 1. However, comparison of the experimental results with the theoretical calculations is complicated by the presence of non-crystallographic symmetry. As a result there are two copies of each TDM contributing to the every absorption band in the sets used to fit the spectra.

For example in the case of the 1706.5 cm^{-1} band that was assigned to PD1 13-1-keto C=O cation vibration, there is a PD1 molecule in both monomers of the crystallographic asymmetric unit,

each with its distinct TDM directions. Thus the experimental dichroic ratio *R* for this band will reflect the direction of the vector sum of the two TDMs.

Due to this complication, further analysis had to rely on the numerical procedure that is described below. Briefly, for each vibrational band, the calculation looked for all possible orientations of TDM that satisfies two conditions. First, the angle between the two copies of the same TDM had to be equal to the equivalent angle from DFT calculations. The second condition required that the *R* (see main text Methods section) for the sum of the TDMs must be equal to the experimental value.

The result of this analysis is presented in Supplementary Figure 18, 19 and 20 for 1706.5 cm^{-1} and 1701 cm^{-1} bands that are assigned correspondingly to PD1 13-1-keto C=O cation and Pheo 13-2-ester C=O anion vibrations. The results show that, even in the presence of non-crystallographic symmetry, an analysis of the three-dimensional orientations of vibrational TDM is possible.

<u>Summary of theory for exciton dynamics and trapping by electron transfer for the crystallographic case</u>

The relative contributions $\Delta_i(t) = N_i(t)p_i$of the pigments to the dichroic absorbance A_{BC} of the pump-probe signal depend on the polarization of the excitation laser and become time-dependent because of energy transfer between the pigments. These dependencies are included in the time-dependent populations $N_i(t)$ofelectronically excited pigment states.

In order to calculate $N_i(t)$ we have to describe the absorption of an ultrashort optical pulse by the core complex that is oriented according to the crystal considered. Because of excitonic couplings between electronic transitions of different pigments the excited states of the core complex are partially delocalized. The delocalization is limited by differences in local optical transition energies and by static and dynamic disorder, caused by slow and fast protein dynamics, respectively. The differences in local optical transition energies are caused by the different protein environments in the different binding sites of the pigments, therefore these energies are often termed site energies. Protein dynamics that is slow compared to excited state lifetimes of the pigments (fs to ns) causes static disorder in site energies and is included by a Monte Carlo average that takes into account a Gaussian distribution function for the site energies. Any fast protein dynamics causes a homogeneous broadening of optical lines and also limits the extent of exciton delocalization. These dynamic localization effects are included in the present theory implicitly by introducing exciton domains of strongly coupled pigments and allowing exciton delocalization to occur only within these domains, as described in detail previously[11,12] . A delocalized exciton state $|M_d\rangle = \sum_{m_d} c_{m_d}^{(M_d)}|m_d\rangle$ is given as a linear combination of localized excited states $|m_d\rangle$ where pigment $m_d$is excited and all other pigments in this domain are in their electronic ground state. The initial populations $P_{M_d}^{(d)}(0)$of the exciton state $|M_d\rangle$in a given domain *d* are defined by the absorption of an ultrashort pump pulse that is assumed to have a Gaussian shaped envelope of FWHM $2\tau_p\sqrt{2\ln 2}$, a carrier frequency Ω, and a polarization along the unit vector $\vec{e}_{pu}$ [12],

$$P_{M_d}^{(d)}(0,\vec{e}_{pu}) \propto \left|\vec{e}_{pu}\cdot\vec{\mu}_{M_d}\right|^2 \int_{-\infty}^{\infty} d\tau e^{-i\left(\Omega-\omega_{M_d}\right)\tau}\, e^{G_{M_d}(t)-G_{M_d}(0)} e^{-\tau^2/(4\tau_p^2)} \quad (1)$$

Where $\vec{\mu}_{M_d} = \sum_{m_d} c_{m_d}^{(M_d)} \vec{\mu}_{m_d}$ is the transition dipole moment for excitation of exciton state $|M_d\rangle$from the electronic ground state, which is obtained as a linear combination of local transition dipole moments $\vec{\mu}_{m_d}$. The frequency ω_{M_d}denotes the transition frequency between the minima of the potential energy surfaces of the ground state and of exciton state $|M_d\rangle$. The function $G_{M_d}(t)$describes excitation of the vibrational sideband of the exciton state $|M_d\rangle$ and is related to the mutual shift of the PES of this exciton state with respect to the PES of the ground state. It is determined by the spectral density $J(\omega)$of the exciton vibrational coupling, the Bose-Einstein distribution function of vibrational quanta and the exciton coefficients $c_{m_d}^{(M_d)}$ [12]. $J(\omega)$ was extracted from experimental line narrowing data, as described before[12]. Please note that in Ref. 12 an average over random orientations of complexes in the sample has been carried out in the calculation of the initial populations $P_{M_d}^{(d)}(0)$. In the present case such an average is not needed since the complexes are oriented in a well-defined way in the crystal. The scalar product $\vec{e}_{pu}\vec{e} \cdot \vec{\mu}_{M_d}$ between the polarization vector of the pump pulse and the exciton transition dipole moment determines the probability of excitation. In the experiments $\vec{e}_{pu}\vec{e}$ was either oriented along the crystallographic b or c axis.

In order to obtain the exciton energies and exciton coefficients an exciton matrix is diagonalized for every exciton domain d. The exciton matrix contains in the diagonal the site energies of the pigments, that is the transition energies at which the pigments would absorb light if they were not coupled excitonically, and in the off-diagonal the excitonic couplings between pigments. The latter were obtained in Ref. 11 from the 1.9 Å resolution crystal structure[8] by applying the Poisson-TrEsp method. We use the site energies obtained in Ref. 11 from fitting optical spectra of the core complex and its subunits.

After creation of the initial populations of exciton states by the optical pulse, the excitation energy can relax within the exciton domains, it can be transferred between different domains, and it can be trapped by charge transfer in the reaction center domain. A system of master equations is solved to obtain the time-dependent exciton state populations $P_{M_d}^{(d)}(t)$, taking into account these different transfer processes

$$\frac{d}{dt} P_{M_d}^{(d)}(t) = -\sum_{c,N_c} \left(k_{M_d \to N_c} P_{M_d}^{(d)}(t) - k_{N_c \to M_d} P_{N_c}^{(c)}(t) \right) - \delta_{d,RC}\, k_{M_{RC} \to RP1} P_{M_{RC}}^{(RC)}(t) \quad (2)$$

In the above master equation the rate constant $k_{M_d \to N_c}$ for $d = c$ describes exciton relaxation between two delocalized states $|M_d\rangle$ and$|N_d\rangle$ in the same exciton domain and for $d \neq c$ excitation energy transfer between delocalized state $|M_d\rangle$in one domain and delocalized state $|N_c\rangle$ in another domain. The former rate constant is taken from Redfield theory and the latter from generalized Förster theory, as described in detail in Ref. 12. In the reaction center domain (d = RC) the trapping of excitation energy by primary charge transfer is described by the rate constant

$$k_{M_{RC} \to RP1} = \left| c_{Chl_{D1}}^{(M_{RC})} \right|^2 k_{\text{intr}} \quad (3)$$

where$\left| c_{Chl_{D1}}^{(M_{RC})} \right|^2$denotes the probability that the primary electron donor $Chl_{D1}$is excited in exciton state $|M_{RC}\rangle$ and $k_{\text{intr}}$is the intrinsic rate constant for primary charge transfer between the excited primary electron donor Chl_{D1}^* and the primary electron acceptor $Pheo_{D1}$

$$Chl_{D1}^{*}Pheo_{D1} \xrightarrow{k_{intr}} Chl_{D1}^{+}Pheo_{D1}^{-}$$

In the calculations we take $k_{intr} = (6\,ps)^{-1}$, as has been estimated from the fluorescence decay for closed reaction centers in Ref.12. Please note that because of the large free energy difference of the initial charge transfer reaction $\Delta G_{intr}^{(0)} \geq$ 175 meV, as has been estimated in Ref. 12, back electron transfer from the radical pair state has practically no influence on the decay of excited states in the time interval considered here (see Fig. 3 in the supporting online information of Ref. 12). Therefore, this back electron transfer is neglected and no further radical pair states need to be included in the present calculations. From the populations $P_{M_d}^{(d)}(t)$ of exciton state $|M_d\rangle$ in a given exciton domain d, we obtain the local population $N_i(t)$of any pigment *i* in that domain by

$$N_i(t) = \left|c_i^{(M_d)}\right|^2 P_{M_d}^{(d)}(t) \tag{4}$$

The VIS/IR pump-probe signal in Figure 1 E/F of the main text is obtained from these local populations as

$$\Delta OD \sim N_i\left(t, \vec{e}_{pu}\right)\left|\vec{e}_i^{(keto)} \cdot \vec{e}_{pr}\right|^2 \tag{5}$$

where the polarization vectors $\vec{e}_{pu}$of the VIS pump pulse and $\vec{e}_{pr}$of the IR probe pulse are taken at all possible combinations of the b- and c-crystallographic axes and $\vec{e}_i^{(keto)}$is the unit vector along the vibrational keto transition dipole moment of the pigments. Please note that $N_i\left(t, \vec{e}_{pu}\right)$ refers to the local excited state population of pigment i after optical excitation with a pump pulse that is polarized along $\vec{e}_{pu}$.

In order to see how the results change if the system would behave like suggested by the ERPE model, we have artificially increased the inter-domain exciton transfer rates by a factor of 50 and decreased the free energy difference of the initial charge transfer reaction to $\Delta G_{intr}^{(0)} =$ 0.02 eV, which makes this reaction reversible, that is we have

$$Chl_{D1}^{*}Pheo_{D1} \leftrightarrow Chl_{D1}^{+}Pheo_{D1}^{-}$$

where the ratio of forward and backward rate constant $k_{intr}/k_{intr-back}$ equals exp($\Delta G_{intr}^{(0)}$/kT) and we slightly decreased the intrinsic rate constant to $k_{intr} = (8.8\,ps)^{-1}$.

In order to allow for an irreversible trapping of excitation energy the secondary electron transfer step

$$P_{D1}Chl_{D1}^{+}Pheo_{D1} \leftrightarrow P_{D1}^{+}Chl_{D1}Pheo_{D1}^{-}$$

was included with a larger free energy difference $\Delta G_{RP1\rightarrow RP2}^{(0)} = 0.138$ eV and a forward rate constant $k_{RP1\rightarrow RP2} = (6\,ps)^{-1}$, where the back transfer rate is given as $k_{RP2\rightarrow RP1} =$ exp$(-\Delta G_{RP1\rightarrow RP2}^{(0)}/kT)k_{RP1\rightarrow RP2}$.

All these charge transfer parameters were chosen such that for this fictitious ERPE type system, the resulting fluorescence decays in exactly the same way as in our original structure-based model, as shown in the upper part of Supplementary Figure 8.

Hence, without structural information it would not be possible to distinguish between the two models solely from the fluorescence decay. In contrast, the calculation of VIS pump- IR probe spectra in the middle and bottom part of Supplementary Figure 8 reveals significant differences of the two models. Due to the faster exciton transfer in the ERPE model, the system "forgets" the polarization of the initial excitation much faster than in the structure-based model. Consequently the anisotropy of the pump-probe signal is practically absent in the ERPE model but clearly visible in the structure-based model. By comparing the calculated with the measured pump probe signals (main text Figure 1 C and D) it becomes clear that only the structure-based transfer to the trap limited model can at least qualitatively explains the experimental data.

A word of caution should be added to the above calculations. A rescaling of the energy transfer times by a factor of 50 is, of course, not in agreement with the structure of photosystem II. Therefore, we have to consider the ERPE type dynamics as that of a fictitious system with a different structure. In the absence of any crystal structure information the decay of the fluorescence alone could not distinguish between the two models, but the present polarization resolved VIS/IR experiment on oriented single crystals could, as our calculations demonstrate.

A quantitative difference between calculation and experiment is that in the intermediate 5-20 ps time range the experimental signals decays faster than the calculated one.

Since for the present pump intensities on average 2.5 photons are absorbed per core complex, as has been estimated in the main text, the data are influenced also by exciton-exciton annihilation effects not included in the theoretical analysis so far. The Coulomb coupling between exciton domains that are both optically excited couples the transition between the one-exciton manifold and the two exciton manifold of one domain to the transition from the one exciton state to the ground state of the other domain. The population of the doubly excited exciton manifold afterwards non-radiatively decays back to the one exciton domain, caused by intra domain exciton-exciton annihilation and overall an excitation has been quenched. If initially the same exciton domain is doubly excited optically, it gets quenched directly by intra domain exciton-exciton annihilation. In the following we will take into account intra-domain exciton-exciton annihilation implicitly, by assuming that it is fast compared to inter-domain exciton relaxation and provide an explicit description of the latter. In addition, we will use the fact that intra-domain exciton relaxation is fast compared to inter-domain exciton transfer, by assuming that inter-domain exciton transfer and inter-domain exciton annihilation starts from equilibrated intra-domain exciton states. In this case the inter-domain rate constant. $k_{d \to c}$ for transfer between exciton domains *d* and *c* follows as

$$k_{c \to d} = \sum_{M_c, N_d} f(M_c) k_{M_c \to N_d} \quad (6)$$

where $f(M_c)$ describes the equilibrium population of intra-domain exciton state $|M_c\rangle$ and $k_{M_c \to N_d}$ is the generalized Förster rate constant for inter-domain exciton transfer between the *M*th exciton state of domain *c* and the *N*th exciton state of domain *d*. Exciton-exciton annihilation process occurring between domains c and d, where an exciton in domain c is annihilated is approximated by the rate constant

$$k_{cd \to d} = \sum_{M_c, N_d, 2K_c} f(M_c) f(N_d) k_{M_c N_d \to 2K_c} \quad (7)$$

where $k_{M_c N_d \to 2K_c}$ is the rate constant for exciton fusion between the *M*th exciton state of domain *c* and the Nth exciton state of domain *d* giving rise to the creation of the *K*th exciton state in the two-exciton manifold of domain *c*. This process is considered to be the rate limiting step of the

exciton-exciton annihilation. In complete analogy to the Generalized Förster rate constant $k_{M_c \to N_d}$for transfer between one-exciton states in different domains we obtain for the rate constant $k_{M_c N_d \to 2K_c}$

$$k_{M_c N_d \to 2K_c} = \frac{2\pi}{\hbar^2} |V_{M_c \to 2K_c, N_d \to 0}|^2 O_{M_c \to 2K_c, N_d \to 0} \quad (8)$$

where $O_{M_c \to 2K_c, N_d \to 0}$ is the spectral overlap between the lineshape functions of the $M_c \to 2K_c$ excitation in domain c and the $N_d \to 0$ transition in domain *d*, and $V_{M_c \to 2K_c, N_d \to 0}$denotes the excitonic coupling between the involved electronic transitions in the two domains that can be expressed in terms of the excitonic couplings between individual pigments and coefficients of one-exciton states $|M_c\rangle = \sum_{m_c} c_{m_c}^{(M_c)} |m_c\rangle$ and two-exciton states $|2K_c\rangle = \sum_{m_c,k_c}^{k_c<m_c} c_{m_c k_c}^{(2K_c)} |m_c k_c\rangle + \sum_{m_c} c_{2m_c}^{(2K_c)} |2m_c\rangle$, where $|m_c\rangle$ denotes a singly excited state that is localized at the *m*th pigment in domain *c* , $|m_c k_c\rangle$ is a doubly excited state localized at the *k*th and the *m*th pigments, and in the state $|2m_c\rangle$ the *m*th pigment of domain *c* is doubly excited.

The two-exciton coefficients are obtained by diagonalizing the two-exciton matrix that contains in the diagonal the site energies $E_m^{(c)} + E_k^{(c)}$ of intermolecular two-exciton states $|m_c k_c\rangle$ and the site energies $E_{2m}^{(c)}$ of the intramolecular two exciton states $|2m_c\rangle$ which we approximate as $E_{2m}^{(c)} \approx 2E_m^{(c)}$.

The inter-domain exciton fusion coupling is given as

$$V_{M_c \to 2K_c, N_d \to 0} = \sum_{m_c,n_d} c_{m_c}^{(M_c)} c_{n_d}^{(N_d)} \left(\sum_{k_c}^{k_c<m_c} c_{m_c k_c}^{(2K_c)} V_{n_d,k_c} + \sum_{k_c}^{k_c>m_c} c_{k_c m_c}^{(2K_c)} V_{n_d,k_c} + c_{m_c m_c}^{(2K_c)} V_{n_d, m_c \to 2m_c} \right) \quad (S9)$$

The excitonic coupling V_{n_d,k_c} describes the coupling of the ground-to-first excited state transition densities of pigment *n* in domain *d* and pigment *k* in domain *c* and $V_{n_d, m_c \to 2m_c}$ that of the transition densities of the the ground-to-first excited state of the *n*th pigment in domain *d* and the first-to-higher excited state transition of pigment *m* in domain *c*. For simplicity, we assume $V_{n_d, m_c \to 2m_c} \approx V_{n_d, m_c}$ and we consider the upper limit of the rate constant $k_{M_c N_d \to 2K_c}$ for exciton-exciton annihilation in Eq. (8) by assuming perfect overlap of lineshape functions, that is, we set the overlap integral $O_{M_c \to 2K_c, N_d \to 0}$ to unity (1 fs).

In the limit of fast intra-domain exciton relaxation the populations of exciton states are Boltzmann equilibrated, that is, we have

$$P_{M_d}^{(d)}(t) = P_d(t) f(M_d) \quad (10)$$

for the singly excited states of the complex, containing the Boltzmann factors *f*, and

$$P_{M_c,N_d}^{(c,d)}(t) = P_{cd}(t) f(M_c) f(N_d) \quad (11)$$

for the doubly excited states that are localized in domains *c* and *d*. The singly-excited domain populations $P_d(t)$ fulfil the following master equation

$$\frac{d}{dt}P_d(t) = -\sum_c \left(k_{d\to c}P_d\ (t) - k_{c\to d}P_c\ (t) - k_{cd\to d}P_{cd}(t)\right) \quad (12)$$

where $k_{d\to c}$denotes the inter-domain exciton transfer and includes now also primary charge transfer in the case that *d* equals RC and *c* equals RP1. As before, we neglect charge back transfer, that is, $k_{RP1\to RC} = 0$. As a new term, not included so far we have the population transfer from the doubly excited state localized at domains c and d to the singly excited state localized at domain d, described by the exciton annihilation rate constant $k_{cd\to d}$.

The population transfer in the doubly excited state manifold is obtained from the master equation

$$\frac{d}{dt}P_{cd}(t) = -\left(\sum_g\left(k_{c\to g} + k_{d\to g}\right) + k_{cd\to d} + k_{cd\to c}\right)P_{cd}(t) + \sum_g \left(k_{g\to c}P_{gd}(t) + k_{g\to d}P_{cg}(t)\right) \quad \text{(S13)}$$

As initial condition we have $P_d(0) = \sum_{M_d} P_{M_d}^{(d)}(0)$ and $P_{cd}(0) = P_c(0)P_d(0)$ with the initial exciton populations $P_{M_d}^{(d)}(0)$ given in Eq. (1).

The annihilation rate constant $k_{cd\to d}$ in Eqs. (12) and (13) so far has been only defined for exciton domains *c* and *d*. Its known, however, that the chlorophyll cation (Chl^+) as it occurs in the radical pair states RP_i in the reaction cente ris an efficient quencher of excitation energy[13]. Assigning a microscopic rate constant for this process would require to assign transition dipole moments for the absorption of Chl^+. Here, we will implicitly take into account this quenching by assuming that it occurs after the excitation energy has been transferred to the RC. Since the bottleneck of this reaction is the transfer to the RC we can approximate the rate constant $k_{dRP_i\to RP_i}$by the rate constant $k_{d\to RC}$for excitation energy transfer from the antenna domain *d* to the respective reaction center, where the radical pair state is located.

We have checked first our assumption of fast intra-domain exciton relaxation by comparing the annihilation free dynamics with and without using this approximation. The resulting pump-probe curves reveal good quantitative agreement for delay times larger than 1 ps. Hence it is justified to use this approximation in the calculation of exciton-exciton annihilation.

The VIS/IR pump-probe spectra obtained for pump-polarization along the crystal b- and c-axes are compared in Supplementary Figs. 9 and 10 to the annihilation free cases. The overall decay of the signals for the different probe polarizations is very similar with and without including annihilation. The annihilation process seems to accelerate the decay of the signals somewhat in the 2-20 ps time window. This time window corresponds to excitation energy transfer times between different domains in the same antenna subunit CP43 or CP47. After 20 ps the decay of the pump-probe signals is very similar, with and without annihilation, but the signal calculated with annihilation has a 20 % smaller amplitude due to the earlier annihilation processes.

A qualitative estimation of the decrease in signal amplitude at late times due to exciton-exciton annihilation processes can be obtained in the following way. We first note that due to the large distance between the CP43/47 subunits and the RC at low light intensities the bottleneck for the decay of excited states is the transfer between these subunits and the RC [Ref 11]. Since also the distance between the CP43, between the CP47 and between the CP43 and the CP47 subunits in PS2 dimers are large, exciton annihilation occurs predominantly between exciton domains

located in the same antenna subunit. In Supplementary Fig. 11 the different realizations of multi-excitation states in the antenna subunits of PS 2 dimers are illustrated. For two excitations the latter might be located either in the same subunit or in two different ones. There are 4 microscopic realizations of the first type and six of the second one (as noted in brackets below the respective illustrations). Only the first type is influenced by exciton-exciton annihilation. The second type decays by transfer to the RC and electron transfer, since the inter-subunit distances are large. Since the inter-domain distances within a subunit (CP43 or CP47) are small compared to the distances between these subunits and the RC, two excitations present in the same subunit will react fast by annihilation and a single excitations is left that is afterwards quenched by transfer to the RC. Hence, in a subunit with multiple excitations only one will contribute to the amplitude of the slow 60 ps decay process in the experiment, reflecting excitation energy transfer from an antenna subunit to the RC. Hence, concerning the relative amplitude η of the slow decay process with and without annihilation for a given number of excitations, we may just count the subunits that have at least one excitation and divide by the number of excitations. Taking into account the different statistical weights of the different types of multi-excitation states in Supplementary Fig. 11, we obtain for the two antenna excitation states

$$\eta_{2\mathrm{exc}} = \frac{4 \times 1 + 6 \times 2}{10 \times 2} = 0.8$$

in very good agreement with the relative amplitude of the signal at large delay times in Supplementary Figure 9 obtained from our microscopic simulations with and without taking into account exciton-exciton annihilation of the two-excitation states. Since the inclusion of more than two excitations in these calculations increases the numerical costs dramatically, we have not taken into account these states so far. Nevertheless, our simple statistical model introduced above can be used to obtain an estimate for the change in relative signal amplitude at large times.

In the case of three antenna excitations we get as a new state that with three excitations in the same subunit. As in the case of the two excitation states we expect efficient quenching of all but one excitation before excitation energy transfer to the RC takes place. Therefore, we obtain

$$\eta_{3\mathrm{exc}} = \frac{4 \times 1 + 12 \times 2 + 4 \times 3}{20 \times 3} = 0.67$$

In complete analogy the relative amplitudes of the slow decay with and without annihilation due to contributions of the 4 and 5 excitation states are given as

$$\eta_{4\mathrm{exc}} = \frac{4 \times 1 + 12 \times 2 + 6 \times 2 + 12 \times 3 + 1 \times 4}{35 \times 4} = 0.54$$

and

$$\eta_{5\mathrm{exc}} = \frac{4 \times 1 + 12 \times 2 + 12 \times 2 + 12 \times 3 + 12 \times 3 + 4 \times 4}{56 \times 5} = 0.50$$

We have marked the expected signal due to the different number of excitations present in the annihilation process as circles at a large time (110 ps) in Supplementary Fig. 9. The relative weight for different number of excitations is determined by the intensity of the pump-pulse applied in the experiment. For the present experiment an average of 2.5 absorbed photons per PS 2 dimer has been estimated in the main text. Hence, it can be expected that at least the three excitation states will have a significant population. Since for larger number of excitations the

average distance between excited domains within one subunit decreases, we expect, besides the smaller amplitude of the signal at large delay times between pump- and probe pulse, discussed above, also faster exciton-exciton annihilation. The larger weight of the shorter annihilation times will lead to an earlier decrease of the signal that will bring the latter closer to the experimental curves of the main text.

Finally, we have investigated an alternative set of site energies of the CP47 pigments, suggested very recently by Hall et al.[14] on the basis of fits of linear optical spectra including linear absorption, linear dichroism, circular dichroism and circularly polarized luminescence (CPL). The most striking new result from this study is that Chl 11 instead of Chl 29 of the earlier studies is suggested to be the lowest energy pigment in CP47. In case of CP43 the site energies of Shibata et al. [12] were found to explain the linear spectra, including the newly measured CPL[14]. We have combined the Shibata et al.[12] site energies for the reaction center pigments and the ones in CP43 with those suggested by Hall et al.[15] for CP47 and recalculated the pump-probe decays with and without including exciton-exciton-annihilation for pump-polarization in B-direction as shown in Supplementary Figure 12. The overall decay of the pump-probe spectra is somewhat slower than the one obtained for the site energies of Shibata et al. in Supplementary Fig. 9, but the overall behavior is qualitatively very similar in the case of the pump pulse polarization along the crystal b axis. For pump pulse polarization along the c-axis the signals for the two probe polarizations cross at about 5 ps and the difference between the two decays on a similar time scale as for the first pump polarization. It is interesting to note that such a crossing of pump-probe signals also occurs in the experiment, but for pump polarization along the crystal b-axis (Figure 1C of the main text).

As before, including annihilation leads again to a faster decay for intermediate times (2-20 ps) that brings the signal somewhat closer to the experimental data. As before one may well expect that the agreement between theory and experiment will increase further by including larger number of antenna excitations in the calculations.

Analysis and modeling of the experimental data

The selection of the crystal to be used in the analysis was performed as follows. For each probe orientation, all available crystal measurements were averaged. Resulting spectra were visually compared to the spectra of each individual crystal. The crystal that was closest to the averaged spectrum was chosen as the most representative and was used in the following analysis. The available data consisted of the pump probe spectra for four polarization combinations. Both pump and probe polarizations could be oriented along either B or C axes of the crystal (see discussion of crystal morphology below). Thus, the time resolved change in absorbance along B and C axes was measured for two pump orientations. Initially the fitting procedure was performed just for one combination of pump and probe orientations. The obtained fit parameters were then used for other polarization combinations, but were kept fixed to facilitate comparison of the measurements. A fine grid of delay points was acquired around presumed zero delay between pump and probe point. This allowed determining “time zero” precisely. After the “time zero” was selected, the time scale was adjusted and data at negative delays was discarded. An additional negative delay point at -200 ps was acquired as a control for noise level of the system and to check for presence of long lived states. No such states were observed in this experiment. Thus the useful experimental data consisted of difference absorption spectra measured at 15

delay points ranging from 0 ps to 1.5 ns for each probe orientation. The data was analyzed using Global Analysis Toolbox[16]. Singular value decomposition[17] showed three significant singular values corresponding to three components in the data. This contrasts slightly with measurements of the liquid PSII sample where four significant components were observed (See also Ref. 7, 2).Thus a global fit was performed using sequential model with three increasing time constants. This resulted in three spectra each associated with a decay constant of 3 ps, 61.5 ps and 832 ps correspondingly. The spectra are shown in the Supplementary Figure 14 for all possible pump and probe orientations.

Energy Transfer spectra fitting

The spectra corresponding to 61.5 ps were illustrative of the energy transfer from the photosynthetic antennae to the reaction centre. At this time scale the spectral region from 1600 cm^{-1} to 1700 cm^{-1} is dominated by the ground state bleach and induced absorbance bands of chlorophyll 13-1-keto C=O vibration. The exact position of the band is affected by the environment and the interactions of the corresponding chlorophyll molecule. The 61.5 ps spectra were modelled using three pairs of negative and positive bands. The lowest energy bleach was paired with the lowest energy induced absorption and so on. Thus three different groups of Chla molecules are assumed to be present in the sample. The position of each of the bleach and induced absorption was determined approximately and was allowed to vary by ±7 cm^{-1} during the fitting. In addition to the Chla 13-1-keto C=O bands several more bands were used in the modelling, mostly in the lower energy region (<1610 cm^{-1}). The assignment of these bands is uncertain and they were not used in the following analysis. Furthermore three weak narrow bands were added to the model in the 1657 – 1672 range. These bands most likely correspond to the absorption of the emerging charge separated state as they become more prominent in the 832 ps spectrum. A further restriction was implemented to the amplitudes of the Chla bands. The anisotropy of the paired negative and positive bands was kept equal. This meant that during the fitting procedure, the same parameter corresponding to the angle was used for both ground state bleach and induced absorption. The fitting was performed using Pattern Search algorithm from Matlab Global Optimization Toolbox. The fit of the 61.5 ps spectrum and the corresponding bands are presented in the Supplementary Figure 16.

Charge separated spectra

The spectrum corresponding to decay constant 832 ps mainly represents the absorption of the charge separated state in the reaction centre. The state consists of $PheoD1^-$ anion and $P680^+$ cation [7]. Pawlowicz and co-authors noted that it is not expected for Q_A to contribute to the charge separated IR spectrum, because due to the relatively high illumination levels, Q_A is in the reduced state[7,18]. Modelling of this spectrum is rather complicated, because of several overlapping positive and negative bands. To aid the modelling of the crystal spectra, FTIR spectra of $Pheo^-$/Pheo and $P680^+$/P680 as well as the pump probe spectrum of the liquid PSII sample were used. As the vibrational spectra of different PSII preparations can vary, only the FTIR spectra from the *T. elongatus* with gene PsbA1 were used in further analysis. The $Pheo^-$/Pheo FTIR spectrum was provided by the authors of [10] and the $P680^+$/P680 spectrum by the authors of [9]. The liquid sample pump probe spectra were obtained with the current setup and in identical conditions as in the crystal spectra case. Furthermore, the focus of the following analysis was restricted to the 1690-1730 cm^{-1} region as it is well investigated using both FTIR and ultrafast spectroscopy methods.

Assignments

For mid-IR TRIR of PSII, the most investigated spectral region is 1650-1750 cm^{-1} range. This is due to the fact that it contains absorption lines corresponding to the keto and ester C=O vibrations of Chla and Pheo molecules. As these vibrations are usually localized on a single molecule and the centre frequency of each transition is indicative of the environment the molecule is in, there is a potential to track each excitation state of each separate molecule and monitor its change in time. However, protein amide I band can also contribute to the absorption spectrum in this region. Below a brief review of the vibrational band assignment in this region is presented.

One of the first analyses of possible band assignments of P680+/P680 spectrum in this region was carried by Noguchi et al.[19]. The assignment of the bands in FTIR difference spectrum of PSII RC sample from spinach was made by comparing it with resonant Raman (RR) spectra of the same sample. Based on this comparison, the authors identified the negative bands at 1679 and 1704 cm^{-1} in their spectrum as belonging to the keto C=O modes of neutral Chl or Pheo molecules. Furthermore, based on structural information, they proposed that 1704cm^{-1} band corresponds to both 13-1-keto C=O vibrations of P680, but the authors also noted that this assignment was not conclusive. The bands at 1711 and 1729 cm^{-1} were proposed as candidates for 13-1-keto C=O modes of P680+. A strong negative band at 1654 cm^{-1} was assigned to amide I vibration because there was no corresponding band in the RR spectrum. Okubo et al. [9] measured P680+/P680 FTIR difference spectra for several different PSII preparations, namely spinach PSII membranes, *T. elongatus* core complexes and spinach reaction centres. Negative peaks at 1701 and 1680 cm^{-1} were consistent between all three preparations with 1701 showing much bigger amplitude than 1680 cm^{-1}. The authors assigned the stronger band to 13-1-keto C=O mode of both neutral PD1 and PD2. Their argument against assigning one of these modes to 1680 cm^{-1} band is based on missing corresponding equally shifted positive band. Furthermore, the authors claim that peak location would indicate highly polar environment or a weak hydrogen bond and they did not observe this situation in X-ray crystallographic structure. The positive peaks at 1711 and 1724 cm^{-1} were assigned to $P680^+$ keto modes. The authors note that the two peaks visible in membrane and core complex spectra merge into one in the reaction centre spectrum. They explain this fact by the charge redistribution between PD1 and PD2 in this sample compared to the other two. The negative band at 1736 is assigned to 13-3-ester C=O vibration of neutral P680 and positive bands at 1743 and 1750 cm^{-1} to cation P680+. Di Donato et al. [17] also reported FTIR difference spectrum of PSII core complexes from Synechocystis cyanobacterium. In contrast to previous findings, the negative bands at 1681 and 1699 cm^{-1} appeared to be of the same magnitude. This lead the authors to suggest that 1681 cm^{-1} band should be assigned to PD2 molecule. From the X-ray crystallographic structure, they suggest that particular hydrogen bond could be formed to lower the frequency of the absorption band. Furthermore, the same paper reported the FTIR spectrum of a mutant which was known to change charge distribution in $P680^+$. This mutation seem to affect not only 1709 and 1724 cm^{-1} bands usually assigned to the cation 13-1-keto C=O mode, but also both 1699 and 1681 cm^{-1} bands. The authors suggested that this points to the fact that 1681 cm^{-1} also corresponded to P680 13-1-keto C=O mode. The most thorough study aimed at determining the assignments of PSII vibrational bands was performed by Romero et al.[20]. First a global N^{15} labelling of PSII reaction centre from spinach was performed. As protein modes were expected to downshift upon isotope exchange, this allowed identifying which FTIR bands belonged completely or were coupled to protein vibrations. The isotope exchange should not affect the modes corresponding

to Chla or Pheo vibrations as verified by DFT calculations performed by the authors. In the case of P680+/P680 FTIR spectrum, the shift upon N^{15} labelling of the bands in the 1650 – 1750 cm^{-1} region is negligible. The only bands to shift by 1 cm^{-1} were 1720 cm^{-1} (positive) and 1702 cm^{-1} (negative). Thus the contribution of protein absorption in this region is negligible and the bands belong exclusively to P680 or $P680^{+}$. While this confirms previous assignments for the bands in the 1670 – 1750 cm^{-1} region, the bands at 1667, 1652 and 1645 cm^{-1} were also attributed to P680, contrary to the suggestions in earlier studies. However, the authors did not attempt to assign these bands to any specific vibration. Furthermore Romero et al. studied how the P680+/P680 difference spectrum changes for different spinach PSII preparations with varying antenna size, namely membrane, core complex and reaction centre. It was observed that the spectra in the 1650 – 1750 cm^{-1} region were identical for all three cases. This result contradicted the previous result of Okubo et al. [9] that found the doublet at 1711 and 1724 cm^{-1} merge into single band in the case of reaction centre preparation. To check whether this inconsistency was due to the use of different redox mediator, Romero et al. acquired the spectra using several combinations of the mediators, including the one used by Okubo et al. However, the resulting spectra still showed two peaks. The authors conclude that the charge distribution in the case of reaction centre preparations is the same as in more intact preparations. The same result was observed in core complex and reaction centre preparations PSII from Synechocystis cyanobacterium. However, Romero et al. noted that in this case the negative band at 1681 cm^{-1} became much more prominent when compared to the corresponding spectra of spinach preparations. Based on previous assignment of this band to PD2 neutral 13-1-keto C=O mode, the authors suggested that the protein environment of the carbonyl groups of PD1 and PD2 is more asymmetric in this case. Summary of all the P680+/P680 vibration band assignments is presented in Supplementary Table 4.

In the case of Pheo-/Pheo, Shibuya et al. measured the FTIR spectrum for different preparations derived from spinach and *T. elongatus* [10]. They proposed that the neutral state 13-1-keto C=O vibration corresponds to the band at around 1680 cm^{-1} for all preparations. Anion state absorption however is upshifted substantially and the shift depended on the hydrogen bonding strength. The observed band position varied from 1603 to 1587 cm^{-1}. The situation in the 1700 – 1740 region is less clear. Shibuya et al. proposed that there are two sets of Pheo molecules in the reaction centre. The molecules with hydrogen bonding have 13-2-ester C=O neutral state band at 1720 cm^{-1} and the anion state band at 1699 cm^{-1}. On the other hand molecules that are hydrogen bond free have corresponding bands at 1741 and 1727 cm^{-1}. The authors did not determine what could be the cause of this heterogeneity. The previously mentioned study by Romero et al.[20] also investigated Pheo-/Pheo mode assignments. As in the case of P680, global N^{15} labelling was performed and difference FTIR spectra were recorded. It was observed that none of the bands in the 1670-1750 cm^{-1} region shifted upon labelling, indicating that all the vibrational bands in this region belong to Pheo or $Pheo^{-}$. On the other hand bands in the 1640-1670 cm^{-1} region downshifted by 1 cm^{-1} and lead the authors to conclude that these bands belong to amide I modes, perturbed by $Pheo^{-}$ formation.

Furthermore, Romero et al. investigated the effect of hydrogen bonding strength between 13-1-keto carbonyl of Pheo and glutamine residue in position D1-130 for cyanobacterium Synechocystis reaction centre. Three mutants were produced that had different residue in this position. The residues formed hydrogen bonds of different strength, varying from none in the case of the D1-Gln130Leu mutant to strong in the D1-Gln130Glu mutant.

As in previous study by Shibuya et al. [10], the negative band at 1739 cm^{-1} was assigned to free 13-2-ester C=O vibration of Pheo. The authors note, that upon reduction this band downshifts with the size of the shift depending on the amino acid in position D1-130. The second negative band in this region – 1723 cm^{-1} was assigned to hydrogen bonded 13-2-ester C=O vibration of Pheo. The authors propose that Tyr126 could be the candidate for establishing the hydrogen bond with 13-2-ester C=O. However, they note that mutation at D1-130 also affected the position of this band and its relative intensity. Thus the authors suggest that the residue at D1-130 influenced the hydrogen bond network around 13-2-ester C=O of Pheo and maybe even formed a bond itself. The negative band at 1678 cm^{-1} was assigned to 13-1-keto C=O vibration of Pheo molecule confirming previous assignment by Shibuya et al. [10.] Its position was constant for all mutants with varying hydrogen bond strength. However, the anion absorption band shift was very dependent on the bond strength. The positive band was at 1630 cm^{-1} for D1-Gln130Leu that did not form hydrogen bond, 1603 cm^{-1} for wild type and 1587 cm- for D1-Gln130Glu which corresponded to strong hydrogen bond. Consequently the authors proposed that 13-1-keto C=O of Pheo is hydrogen bonded only in reduced state. However, presence of weaker 1630 cm^{-1} band in all mutant spectra could indicate that there were two populations of Pheo molecules, thus confirming the interpretation of 13-2-ester C=O vibration assignment and consistent with suggestions of Shibuya et al. A summary of the Pheo-/Pheo vibration band assignments is presented in Table 4.

Fitting the FTIR spectra

The derivative method[21] was used to identify the band positions in the FTIR spectrum for both $Pheo^-$/Pheo and $P680^+$/P680 spectra. Then a set of Gaussian bands at these positions was used to model the FTIR spectra by varying their widths and positions within several cm^{-1} range. The fit of the FTIR spectra with set of Gaussian bands is presented in the Supplementary Figure 13. As expected in $P680^+$/P680 spectrum the strongest bands belong to 13-1-keto C=O vibrations – negative at 1700 cm^{-1} and two positive at 1706.5 and 1723.5. A small band corresponding to the 13-3-ester C=O vibration was observed at 1742 cm^{-1}. The fit results of the $Pheo^-$/Pheo FTIR spectrum presents a more complicated situation. While the main 13-2-ester C=O bands at 1701 cm^{-1} (positive), 1722 cm^{-1} (negative), 1733 cm^{-1} (positive) and 1741 cm^{-1} (negative) are observed as expected, additional lines had to be added to produce acceptable fit results. The assignment of the added 1692 cm^{-1} and 1714 cm^{-1} bands is currently unknown.

Fitting the 832 ps spectrum

The resulting set of bands served as a starting point for the modelling of the 832 ps spectrum. An additional restriction was introduced concerning the relative amplitude of individual bands. The common amplitude multiplier for all bands coming from the same FTIR spectrum (either Pheo or P680) could vary freely, but the multiplier of each individual band amplitude was restricted to the 0.5 – 2 range. This helped avoiding physically unrealistic modelling results and maintained relative proportionality of the fitted bands. The result of the modelling of charge separated state spectra with the set of bands obtained above is presented in the Supplementary Figure 17. Some of the bands had to be moved relative to their positions in the FTIR spectrum, but the adjustment never exceeded 2 cm^{-1}. This was needed because different conditions for sample preparation in crystal case and better resolution of the crystal spectra. To confirm that the need for band adjustment was not due to crystalline nature of the current sample, a pump probe spectrum of the liquid PSII sample was modelled using exactly the same bands as in crystal case. The result is

shown in Supplementary Figure 13 and clearly demonstrates that a very good fit could be achieved without the need for adjustment of the bands.

The fitted amplitudes for all possible pump and probe orientations are presented in the Supplementary Table 4. A possible assignment of each of the bands is also indicated in the table.

Structural information in charge separated state spectra

It has been demonstrated that structural information can be extracted from IR anisotropy measurements of protein crystals[1]. However in the current case, PSII crystals have thin plate morphology and only one crystal face is available of optical measurements. Due to this fact only results along one crystallographic axis are available. The analysis of the results is further complicated by the presence of non-crystallographic symmetry in the PSII crystals. That means that each unit cell contains two copies of each of the molecules contributing to the IR spectrum. Each of these molecules has different orientations and as a result non-parallel transition dipole vectors. Thus in this case, the task to uniquely identify the molecule or bond responsible for certain band in the spectrum requires the analysis of the noncrystallographic symmetry. Numerical modeling is used to obtain all possible dipole orientations that could reproduce experimental results and the results are compared to the results from DFT calculation.

However before starting the numerical analysis, a discussion of the ways to identify crystal orientation is needed. The PSII crystals grown for this experiment had cuboid morphology with one edge significantly shorter that other two. The thickness of the crystal in this dimension was chosen to optimize its transmission of the IR beam. The shape of the crystal in other dimension formed either a square or rectangle. For this experiment, crystals of latter shape were chosen to facilitate axis identification. While the dimensions of the crystals for pump probe measurements were usually of the order of tens of microns, some of the crystals in the preparation were allowed to grow to the size suitable for X-ray crystallography. Out of those crystals, the ones with rectangular morphology were used for X-ray diffraction measurements and the orientation of their axes was determined. It was consistently observed that the shortest edge of the crystals corresponded to the A axis of the crystal (n=17). The result was less conclusive in the case of the other two dimensions. Although most of the crystals had C axis orientated along the longest edge, some crystals had this orientation reversed. Thus it was concluded that this information cannot be used to determine the axis orientation of the crystals in pump probe measurements. Further spectroscopic information was used to identify the orientation of the C axis. One of the strongest absorption lines in the 1550 – 1750 cm^{-1} region belongs to Pheo 13-1-keto C=O anion state vibration at 1603 cm^{-1}. Both X-ray crystallographic data [3] and DFT calculations indicate that this bond is oriented predominantly along C axis. Therefore, for this band, the absorption along the edge coinciding with the C axis should be significantly stronger than that coinciding with the B axis. This feature used to identify the C axis orientation in the crystals.

Numerical analysis of charge separated data

The experiment provided sample absorbance for the probe polarization orientation along B and C axes. The ratio of the two absorbances depends only on the orientation of the transition dipole projection onto BC plane [1]. However, as mentioned above, in the case of PSII crystal there are two copies of the dipole contributing to each band. Thus the ratio of the absorbances is proportional to the sum of squared BC projections of the two dipoles and this prevents from

obtaining structural information directly. Therefore further analysis was based on a comparison of the experimental results with the structure of PSII reaction centre produced by the DFT calculations.

For all bands that had a vibration assigned to them, the orientation of the two bonds responsible for this vibration was obtained. Next, an assumption was made that the orientation of the transition dipole coincided with that of the corresponding bond. Thus the vector connecting the atoms in the bond was assumed to be parallel to the transition dipole vector. Therefore, each assigned band had two calculated transition dipole vectors and experimentally measured absorption ratio associated with it. To verify the above assumption as well as the assignment of the vibrations, following numerical procedure was performed. While keeping the angle between two copies of the same dipole constant, the pair was numerically rotated to all possible orientations using multiplication by Euler matrix. For each orientation, a ratio of absorbances along B and C axes was evaluated. The orientations, that had this ratio equal to the one observed in the experimental spectrum, were recorded. This resulted in a 3D map of all possible orientations of each dipole in the pair that produce the absorbance ratio observed in the spectrum. Such maps are presented in the Supplementary Figures 18-20 using projection of the sphere on to a 2D plane. Furthermore, the orientation of each of the dipoles as obtained using the structure from DFT calculation is presented in the Supplementary Figure 18-20 as well.

Reproducibility of the results and sample degradation

More than 10 crystals of similar morphology were used in the TRIR measurement. The GA fitting was performed on all of the results obtained with the crystals. All of the resulting spectra had the same spectral shapes and only small differences in the amplitudes. The crystal that we used in the analysis was chosen because it had closest resemblance to the average of all 10 crystals. To illustrate the reproducibility of our experimental results, Supplementary Figure 21 shows the results of two crystals GA fitting. Namely 3 ps and 832 ps component spectra are shown for two probe polarizations.

As far as possible sample degradation is concerned, the signal size was constantly monitored during the measurement as an indication of the sample state. All the data presented in the paper comes from the measurements that showed none or negligible amount of degradation. In the case of the crystal used in the analysis above, 60 scans were performed and averaged. Supplementary Figure 22 shows the signal levels at the beginning and the end of the measurement. It can be clearly seen that no significant change in the shape of the spectrum or signal amplitude was observed in the measurement.

Supplementary References

1. Sage, J. T. *et al.* Infrared protein crystallography. *Biochim. Biophys. Acta* **1814,** 760-777(2011).
2. Kaucikas, M., Barber, J. & Van Thor, J. J. Polarization sensitive ultrafast mid-IR pump probe micro-spectrometer with diffraction limited spatial resolution. *Opt. Express* **21,** 8357 (2013).
3. Loll, B., Kern, J., Saenger, W., Zouni, A. & Biesiadka, J. Towards complete cofactor arrangement in the 3.0 A resolution structure of photosystem II. *Nature* **438,** 1040–4 (2005).
4. Ferreira, K. N., Iverson, T. M., Maghlaoui, K., Barber, J. & Iwata, S. Architecture of the photosynthetic oxygen-evolving center. *Science* **303,** 1831–8 (2004).
5. Born, M. & Wolf, E. *Principles of Optics: Electromagnetic Theory of Propagation, Interference and Diffraction of Light*. (Cambridge University Press, 1999).
6. van der Weij-de Wit, C. D., Dekker, J. P., van Grondelle, R. & van Stokkum, I. H. M. Charge separation is virtually irreversible in photosystem II core complexes with oxidized primary quinone acceptor. *J. Phys. Chem. A* **115,** 3947–56 (2011).
7. Pawlowicz, N. P., Groot, M.-L., van Stokkum, I. H. M., Breton, J. & van Grondelle, R. Charge separation and energy transfer in the photosystem II core complex studied by femtosecond midinfrared spectroscopy. *Biophys. J.* **93,** 2732–42 (2007).
8. Umena, Y., Kawakami, K., Shen, J.-R. & Kamiya, N. Crystal structure of oxygen-evolving photosystem II at a resolution of 1.9 Å. *Nature* **473,** 55–60 (2011).
9. Okubo, T., Tomo, T., Sugiura, M. & Noguchi, T. Perturbation of the structure of P680 and the charge distribution on its radical cation in isolated reaction center complexes of photosystem II as revealed by fourier transform infrared spectroscopy. *Biochemistry* **46,** 4390–7 (2007).
10. Shibuya, Y. *et al.* Hydrogen bond interactions of the pheophytin electron acceptor and its radical anion in photosystem II as revealed by Fourier transform infrared difference spectroscopy. *Biochemistry* **49,** 493–501 (2010).
11. Raszewski, G. & Renger, T. Light harvesting in photosystem II core complexes is limited by the transfer to the trap: can the core complex turn into a photoprotective mode? *J. Am. Chem. Soc.* **130,** 4431–46 (2008).
12. Shibata, Y., Nishi, S., Kawakami, K., Shen, J.-R. & Renger, T. Photosystem II does not possess a simple excitation energy funnel: time-resolved fluorescence spectroscopy meets theory. *J. Am. Chem. Soc.* **135,** 6903–14 (2013).
13. Schlodder E., Cetin M., Byrdin M., Terekhova I. V., Karapetyan N. V., $P700^+$ - and ^{3}P700-induced quenching of the fluorescence at 760 nm in trimeric Photosystem I complexes from the cyanobacterium Arthrospira platensis. *Biochimica et Biophysica Acta (BBA)-Bioenergetics,* **1706**, 53-67 (2005).

14. Hall, J., Renger, T., Müh, F., Picorel, R., & Krausz, E. The lowest-energy chlorophyll of photosystem II is adjacent to the peripheral antenna: Emitting states of CP47 assigned via circularly polarized luminescence.*Biochimica et Biophysica Acta (BBA)-Bioenergetics*, **1857**, 1580-1593 (2016).

15. Hall, J., Renger, T., Picorel, R., Krausz, E. Circularly polarized luminescence spectroscopy reveals low-energy excited states and dynamic localization of vibronic transitions in CP43. *Biochimica et Biophysica Acta (BBA)-Bioenergetics,* **1857**, 115-128 (2016).

16. van Wilderen, L. J. G. W., Lincoln, C. N. & van Thor, J. J. Modelling multi-pulse population dynamics from ultrafast spectroscopy. *PLoS One* **6,** e17373 (2011).

17. Van Stokkum, I. H. M., Larsen, D. S. & Van Grondelle, R. Global and target analysis of time-resolved spectra. *Biochim. Biophys. Acta* **1657,** 82–104 (2004).

18. Di Donato, M. *et al.* Primary charge separation in the photosystem II core from Synechocystis: a comparison of femtosecond visible/midinfrared pump-probe spectra of wild-type and two P680 mutants. *Biophys. J.* **94,** 4783–95 (2008).

19. Noguchi, T., Tomo, T. & Inoue, Y. Fourier transform infrared study of the cation radical of P680 in the photosystem II reaction center: evidence for charge delocalization on the chlorophyll dimer. *Biochemistry* **2960,** 13614–13625 (1998).

20. Romero, E. The Electronic Structure of Photosystem II: Charge Separation Dynamics. PhD disseratation. Vrije Universiteit (2011). http://www.narcis.nl/publication/RecordID/oai%3Adare.ubvu.vu.nl%3A1871%2F19044

21. Giese, A. T. & French, C. S. The Analysis of Overlapping Spectral Absorption Bands by Derivative Spectrophotometry. *Appl. Spectrosc.* **9,** 78–96 (1955).
